# Early Warning Signs of a Mental Health Tsunami: A Coordinated Response to Gather Initial Data Insights From Multiple Digital Services Providers

**DOI:** 10.3389/fdgth.2020.578902

**Published:** 2021-02-10

**Authors:** Becky Inkster, Digital Mental Health Data Insights Group (DMHDIG)

**Affiliations:** Wolfson College, University of Cambridge, Cambridge, United Kingdom

**Keywords:** COVID-19, financial stress, isolation, anxiety, data insights, digital mental health

## Abstract

**Introduction:** The immediate impact of coronavirus 2019 (COVID-19) on morbidity and mortality has raised the need for accurate and real-time data monitoring and communication. The aim of this study is to document the initial observations from multiple digital services providers during the COVID-19 crisis, especially those related to mental health and well-being.

**Methods:** We used email and social media to announce an urgent call for support. Digital mental health services providers (*N* = 46), financial services providers (*N* = 4), and other relevant digital data source providers (*N* = 3) responded with quantitative and/or qualitative data insights. People with lived experience of distress, as service users/consumers, and carers are included as co-authors.

**Results:** This study provides proof-of-concept of the viability for researchers and private companies to work collaboratively toward a common good. Digital services providers reported a diverse range of mental health concerns. A recurring observation is that demand for digital mental health support has risen, and that the nature of this demand has also changed since COVID-19, with an apparent increased presentation of anxiety and loneliness.

**Conclusion:** Following this study, we will continue to work with providers in more in-depth ways to capture follow-up insights at regular time points. We will also onboard new providers to address data representativeness. Looking ahead, we anticipate the need for a rigorous process to interpret insights from an even wider variety of sources in order to monitor and respond to mental health needs.

## Introduction

During the coronavirus 2019 (COVID-19) pandemic, traditional mental health services and related activities declined, in part, due to outpatient clinics being closed to adhere to social distancing requirements, mental health staff redeployment, and inpatient beds being converted into COVID-19 units. As governments attempt to contain the virus, we must mitigate the mental health impact of the pandemic and economic crisis, especially given that pre-COVID-19 predictions already indicated that by 2030, depression will be the leading cause of disease burden globally (1).

During the severe acute respiratory syndrome (SARS) (2002–2004) epidemic, social disengagement, mental stress, and anxiety were associated with increased suicide rates in the elderly population (2). Another study found that 30% of children and 25% of parents who were quarantined or isolated during pandemic diseases met the clinical criteria for post-traumatic stress disorder (3). Data from previous economic depressions and recessions suggest profound increases in substance use disorder, depression, and suicide (4, 5).

In the current pandemic, frontline healthcare workers face the possibility of anxiety and burnout (6, 7) and moral injury (8), alongside fears of becoming ill. This is more pronounced among ethnic minority frontline healthcare workers due to the apparent increased health risks associated with COVID-19 (9). For others living in highly conflicted households, social distancing has meant prolonged social contact and abuse. For example, in the UK, the number of suspected domestic homicide victims more than doubled during the first 3 weeks of the lockdown (10). In France, calls to the national violence against children helpline increased by 89% (11). From an economic perspective, a survey of UK households 3 weeks into the “lockdown” found that 49% of households feel anxious about their finances, rising to 95% among the households experiencing serious financial difficulties (12). A survey conducted in March 2020, just as the lockdown rules were coming into place in the USA, also highlighted higher levels of psychological distress among lower income households (13).

There is a need to obtain more granular and real-time information to help us understand the nature and scale of the mental health crisis. A possible source of this information is the large number of digital mental health services providers used by millions of people globally. These include patient to clinician communication tools, digitally enabled treatments, self-managed care solutions, mental health and well-being apps, online forums, support networks, and digital communities. In addition to this, given the established links between health, social, and economic factors (e.g., (14)), insights should also be obtained from financial services providers and other relevant digital data sources. The potential value of healthcare insights in financial data is already recognized (15, 16), and financial services firms not only are a source of uniquely constructive data on household economies (17) but can also offer possible mechanisms of direct and indirect mental health interventions.

To investigate the impact of COVID-19 on mental health, we set out to collect observations from multiple digital services providers (Supplementary Table 1). To our knowledge, this has never been done at scale before, and we did not know how many providers would respond or what the nature of their data insights might be. With rapid turnaround, a diverse range of providers came forward with collective information sourced from a user base of at least 10 million people, but possibly reaching upwards of 50 million globally.

## Materials and Methods

We used email and social media to announce an urgent call for support to investigate the scale and nature of the mental health impact of COVID-19 ^1^. Starting 6 April 2020, BI sent emails to all speakers who had presented at previous “Digital Innovation in Mental Health” (DIMH) conferences ^2^, as well as to members of the FinHealthTech Consortium ^3^, and also to a much wider digital community *via* LinkedIn, Twitter, and Facebook. Additionally, we encouraged providers and co-authors to ensure that they sought the views of people in their own networks.

We reached out directly to 55 digital services providers. We received confirmation of support to contribute from 53 providers (i.e., a positive response rate of 96%), which consisted of 46 digital mental health services providers, four financial services providers, and three other digital data source providers (*N* = 3). Respondents were asked to provide qualitative and/or quantitative insights with no exchange of data or identifiable information. A list of digital services providers can be found in Supplementary Table 1. This study was purely exploratory. We deliberately did not provide a framework for insights or any analytic specifications (e.g., what specific hypotheses to test). Therefore, all insights should be considered illustrative examples, not primary research.

We asked providers to be compliant with the General Data Protection Regulation (GDPR) and the Data Protection Act 2018 if their users were within Europe. To set a good example of responsible innovation, this document only accepted data insights from providers with clear and accessible privacy policies. Other than these ethical grounds, there were no other exclusion criteria. There were no specific inclusion criteria, but many of the respondents had a pre-existing association with members of the study team, for example, through the annually run conference, DIMH, created by Dr. Becky Inkster^2^.

We deliberately did not select a specific methodology for this study, and we did not test any specific hypotheses. Providers collected very different types of data and analyzed it in their own way using techniques that were appropriate for their data. Trying to develop some common methodologies is a future goal, which will require more time, and increased collaboration between different providers and other stakeholders.

Data insights and draft versions of the paper were shared among all co-authors for feedback, including from people with a range of lived experiences of distress and service use.

## Results

Given the anecdotal nature of many of the insights and the non-systematic way in which providers were chosen, we are reluctant to draw conclusions from the content provided in Supplementary Tables 2, 3. Instead, we summarize some of the more frequent observations reported by providers.

### Intentions

Insights suggest changes in the type of information individuals are seeking or presenting. From Google Trends data, searches for “anxiety symptoms” doubled between the weeks beginning 8 March and 22 March 2020. In a similar timeframe, Mental Health America (MHA) witnessed a 22% increase in the numbers of Generalized Anxiety Disorder 7-item (GAD7) anxiety screens (*N* = 11,033) taken in March 2020 compared with February 2020. Qualitative insights suggest that individuals are seeking practical resources and coping strategies. Participants in the It's Ok To Talk discussion raised questions about anxiety, strategies to manage work, studies, sleep, dealing with domestic violence, and difficult home relationships. Babylon reports that many patients are seeking advice on information about local council support services, seeking advice for activities to keep busy and how to remain healthy, and how to get support to access food and financial concerns. Ieso Digital Health reports up to a third of patients mentioning COVID-19 as a reason for presenting for mental health treatment and also reports a rise in patient worries about viruses, with up to 15% of in-session worries about COVID-19.

### Affiliative Tendencies

Papa reported that 53% of users felt less lonely, and that virtual companions have performed a range of tasks with elderly users (e.g., obtaining medications, online grocery shopping). Peer support specialists are being rapidly trained. Digital Peer Support trained 750 peer support specialists between 10 March and mid-April 2020. Wisdo reported a 283% increase in the numbers of people replying to other people's messages and an increase of 115% in the numbers of people signing up for roles to provide support for others. Mentally Aware Nigeria Initiative (MANI) trained over 200 psychosocial support team specialists/counselors on mental health.

### Support-Seeking

Many providers are experiencing increased support-seeking behaviors. For example, Ieso Digital Health reports an 84% increase in referrals. Vala Health reports a doubled volume of mental health-related consultations with general practitioners (GPs) during the period 10 March to 8 April 2020. By week 4 of the UK lockdown, general health enquiries had returned to almost pre-COVID levels, but mental health consultations continued to rise. National Alliance on Mental Illness (NAMI) reports a 41% increase in demand for HelpLine resources and information. Ieso Digital Health reported an 84% increase in referrals to their 1–1 online cognitive behavioral therapy (CBT) service in the weeks since the lockdown was announced in the UK, relative to the same period in 2019. Wysa witnessed a 77% increase in new users during February to March 2020, as compared with the same period in 2019. MANI recorded the highest number of emergency calls in the month of April. Qualitative insights from Orygen (Australia) revealed that young people report privacy concerns in having telehealth consults with family members in the background.

### Outcomes

Many providers report observations suggesting increased anxiety, uncertainty, loneliness, and loss. MHA reports that 45% of people who took an anxiety screen in March (*N* = 11,033) scored for severe anxiety. In a self-reported questionnaire to members of The Mighty, 89% of members reported that their daily life has been at least somewhat impacted by increased anxiety; 43% say that it has been extremely impacted. This is consistent with reports from Kooth demonstrating increases in sadness (up 161%), health anxiety (up 155%), sleep difficulties (up 90%), concerns over body image (up 43%), eating difficulties (up 31%), loneliness (up 23%), and bereavement (up 20%). The Mental Health Foundation survey reported that respondents felt increasingly lonely, and that this was most pronounced for people aged 18–24 (44%) and 25–34 (35%). Multiple providers report users mentioning their loss of access to care and human support [The Mighty, MeeTwo, Wysa, consultant National Health Service (NHS) nurse].

Qntfy's observations suggest decreased well-being in the general public, and that at times, this has been greater among those who identify as healthcare providers. Unmind and Wysa reported higher anxiety levels in health staff than in the rest of their populations. Sangath reports that community health workers face “fears and insecurities among their patients, as well as added anxieties about the health and well-being of their own children and family members.” CBTClinics report a rise of anxiety and depressive type disorders from people emotionally close to frontline health staff (e.g., parents, spouses, and children).

Other outcomes include increases in reporting of unsafe domestic settings (Babylon, Wysa, Teen Line, Kooth), suicidal risk/ideation (MeeTwo, Qntfy, Mental chat, Beyond Blue, Mumsnet), and sleep disturbances (It's OK to Talk, Kooth, Mumsnet, Qare, BioBeats, Wysa). There have also been increased prescription of anti-depressant medications (Jasvinder Kandola), increased requests for pain killers *via* telehealth (Vala Health), and increased activities on darknet markets mentioning psychiatric medications (The TellFinder Alliance).

### Financial Concerns

Financial insights show an overarching theme of the interrelation between mental health and financial health worries. Three sub-themes emerged from the data: (1) uncertainty and a sense of loss of control particularly “at-risk” individuals and groups; (2) anger, anxiety, and concerns over access to financial support especially those who feel that they are “falling through the gaps”; and (3) negative mental health and/or physical health with financial health outcomes.

The Money and Mental Health Policy Institute survey (*N* = 568) reported a range of concerns by respondents with lived experience of mental health problems about how changes, as a result of COVID-19, might affect their finances: 62% worried about having to access the benefits system, 57% worried about losing their job, and 56% worried about creditors chasing them for money. Tully and OpenWrks Group reported that 81% of self-employed customers (*N* = 650) have declared that they do not have any work coming in due to COVID-19. Furthermore, 50% of their wider sample (*N* = 1,822) have had income reduced, and 19% have lost their income. The Turn2us survey showed that 70% of respondents (*N* = 6,198) who have had employment affected are unable to afford rent or mortgages. An anonymous financial services provider also shared concerns that their on-site cashiers may be vulnerable and distressed by customer behaviors.

Qualitative insights also make it clear both how emotive and tangible the impacts of financial concerns and outcomes are on mental health worries and outcomes. For example, “we are… dead… no money no food… 4 weeks in isolation UC no answers… I have no other way to provide for my children and I don't care about the bills… I will have to go out and improvise something.”; “what about the thousands who started new jobs to better ourselves after the Feb 28th cut off and before the #coronavirus hit the UK but now sit suicidal in the gap entitled to nothing despite being lifelong tax payers? #newstarterjustice #newstarterprotest #newstarterfurlough”; and “got my letter yesterday to tell me it's being taken away. The welfare system has kicked me when I'm down already, made me physically ill & caused a flare up of my health just when I don't need to go to a hospital mid pandemic.”

## Discussion

To our knowledge, this study is the first of its kind to bring together a large number of private organizations, including financial services providers, to share digital data insights about the mental health concerns of millions of people online. Our study is novel and radical because this is the first time that something like this has been achieved in this field. Many people questioned the feasibility of being able to bring together a large group of digital services providers (some of which are in competition with each other) to share their insights. We believe that our study provides a proof-of-concept for the viability of using this approach.

The information that we have quickly compiled has been sourced from different geographies, demographics, and types of digital interactions and provides insights into the diversity of individual mental health needs. During our study, a paper (18) called for mental health monitoring to move beyond NHS linkage, in order to capture the real incidence in the community and embrace new technologies measuring moment-to-moment change. This initial snapshot of data that we collected could help inform future studies, for example, it could help the research community to understand what questions could be asked (especially those without expertise in mental health), to aid in the generation of specific hypotheses, or to help with the formulation of prior probabilities. Additionally, we hope that this study increases the research community's awareness of the digital mental health landscape and the services providers who are currently collecting data, as well as the types of data insights and metrics that they might be able to provide.

We recognize that this study is not rigorous in terms of data collection and methodology. We did not choose these providers in a systematic way. Using data from digital services providers limits our population to people who have access to these digital platforms and many “hidden” populations are not registering in digital spaces. Furthermore, we do not know whether our demographic is representative of any larger population or whether whole-population impacts can be inferred from digital service impacts. In addition, we did not verify the insights shared by providers. This avoided privacy issues, but has the potential to have introduced inaccuracies or biases in the reported information. This study is also unable to characterize mental health problems at a clinical level because most digital providers did not report clinically-validated measurements. The use of digital measurements to monitor mental states and distress is still a developing space.

Prior to this study, we did not know what the response rate would be or what types of insights we would be able to obtain. Developing new methodologies to combine such insights will be a substantial undertaking, which should involve many stakeholders. Developing such methodologies is a future goal, but it was not the purpose of this study.

It is important to note that this study was conducted in the midst of the initial pandemic, a time of significant uncertainty. Between the time of the data gathering for and the publication of this study, there have been countless responses across countries announced and enacted. The insights discussed here capture an important moment in time during the initial pandemic phase and also offer a useful reference for on-going data monitoring and subsequent study follow-ups.

Following this study, we will continue to work with providers to capture follow-up insights at later time points^2^ and onboard new providers to address issues of data representativeness. We will continue to engage with and include people with a range of experiences of distress and service use, so that we are inclusively influenced by their insights and inputs. It will also be important to capture insights that relate to resilience and recovery. An important next step will be to develop rigorous means to bring together public and private sector data to monitor mental health needs in real-time (just as contact tracing is used to manage the viral epidemic). This can fuel research and understanding and help to inform high-quality responses, which can be delivered remotely to those in need on global and local scales.

## Data Availability Statement

The data analyzed in this study is subject to the following licenses/restrictions: we only had access to the data insights provided by digital services providers, we did not access the data. Requests to access these datasets should be directed to becky@beckyinkster.com.

## Ethics Statement

Ethical review and approval was not required for the study on human participants in accordance with the local legislation and institutional requirements. Written informed consent from the participants' legal guardian/next of kin was not required to participate in this study in accordance with the national legislation and the institutional requirements. Digital services providers followed their own in house ethical procedures, terms and conditions and consent procedures for their own data sets.

## Author Contributions

BI formulated the idea, operationalised and co-ordinated the response, inviting co-authors to join, all having different professional and/or lived experiences who have made important contributions in various ways, such as performing literature searches, writing, helping us to connect with digital providers, idea generation, editing, interpretation, etc.

## Conflict of Interest

BI is an advisor to Wysa and TalkLife and has worked with the majority of these providers previously via either her Digital Innovation in Mental Health conferences and/or her FinHealthTech Consortium. KJ, JG, and MJ are employed by MindWave Ventures. AC, RC, TC, JB, VT, SB, and AB are employed by Ieso Digital Health. TP, KG, and ES are employed by Huma Therapuetics. PT is employed by Vala Health. DR and KS are employed by Ooca. AM and AS are employed by Kooth. DR, AE, and JP are employed by Silver Cloud Health and Trinity College Dublin. AB is employed by CBTClinics. EP is employed by Minddistrict. CL is employed by Big Health. FJ and LT are employed by Qare. DP is employed by Huma Therapeutics. AS and HB are employed by Unmind. KS is employed by Expert Self Care Ltd (distrACT app). TB, AM, and IA, Alpha Health, Telefonica Innovation Alpha. LA-P, RD, and SL work with ORCHA. JA RV, SJ, VS, and MK are employed by Wysa. CS is employed by Owlie. GI and AH are employed by Riliv. KC, AL, and KT are employed by Stop Blues. CR is employed by BeyondBlue. ML and CC are employed by Neurum Health. WA-M is employed by Spill. JR is employed by Mumsnet. SR and AC are employed by The Mighty. JR and JD are employed by TalkLife. BG is employed by Wisdo. SG and KC are employed by MeeTwo. SR is employed by MIELI Mental Health Finland Mental-chat & Mental Gaming, Finland. MC is employed by Teen Line, USA. AP is employed by Papa. KD and DG are employed by National Alliance on Mental Illness, USA. TN and MR are employed by Mental Health America, USA. VU and IW are employed by Mentally Aware Nigeria Initiative (MANI), Nigeria. CB, AF-E, GG, DK, KP-J, DJ, CS, CU, and LZ are part of the Young Leaders for the Lancet Commission on GlobalMental Health and Sustainable Development. GC and AF are employed by Qntfy, USA. MH, CD’A, and KA are employed by Money & Mental Health Policy Institute, UK. JK is employed by Turn2Us, UK. LH is employed by IncomeMax, UK. OB is employed by Tully and OpenWrks Group, the team behind Tully. AS is employed by Healthy Virtuoso, Italy. WV is employed by The Mind and Soul Foundation, UK. AT, DS, CD, and AS work for The TellFinder Alliance, USA. SV has received funding from iqvia for toolbox development. TI works for Humanest Care.

## Footnotes

^1^Available online at: https://www.beckyinkster.com/covid19 (accessed May 17, 2020).

^2^Available online at: https://www.beckyinkster.com/summer-2021-conference (accessed May 17, 2020).

^3^Available online at: https://www.beckyinkster.com/fhtc (accessed May 17, 2020).

## Digital Mental Health Data Insights Group (DMHDIG)

Becky Inkster, Wolfson College, Cambridge University, Cambridge, UK & The Alan Turing Institute, London, UK; Ross O'Brien, Central and North West London NHS Foundation Trust, and Healthy London Partnership, NHS, UK; Kate Niederhoffer, Knowable Research, Texas, USA; Niranjan Bidargaddi, College of Medicine & Public Health, Flinders University, Adelaide, South Australia, Australia; Anne-Claire Camille Stona, Lee Kong Chian School of Medicine, Nanyang Technological University, Singapore; Glen Coppersmith, Qntfy, USA; Amanda Towler, The TellFinder Alliance; The TellFinder Alliance, USA; Philip Resnik, Department of Linguistics and Institute for Advanced Computer Studies, University of Maryland, Maryland, USA; Rebecca Resnik, Rebecca Resnik and Associates, Bethesda, Maryland, USA; Maria Liakata, Queen Mary University of London, UK; The Alan Turing Institute, UK; University of Warwick, UK; Helen Barker, London, UK; Abdullahi Abubakar Kawu, Ibrahim Badamasi Babangida University, Lapai, Nigeria, Africa; Karen Machin, School of Health & Social Work, University of Hertfordshire, UK; Survivor Researcher Network, UK; Pattie Pramila Gonsalves, Sangath, India; Sweta Pal, Sangath, India; Swetha Ranganathan, Sangath, India; John A. Naslund, Department of Global Health and Social Medicine, Harvard Medical School, Harvard University, Massachusetts, USA; Jo Robinson, Orygen, Parkville, Melbourne, Victoria, Australia, Centre for Youth Mental Health & The University of Melbourne, Parkville, Melbourne, Victoria, Australia; Munmun De Choudhury, School of Interactive Computing, Georgia Institute of Technology, Atlanta Georgia, USA; Glenn Melvin, School of Psychology, Faculty of Health, Deakin University, Melbourne, Australia; Terry Hanley, University of Manchester, UK; Matthew Jackman, Lived Experience Academic, Western Pacific Region, Global Mental Health Peer Network, Melbourne, Australia; Ed Humpherson, Director General for Regulation, United Kingdom Statistics Authority, UK; Bo Wang, Department of Psychiatry, University of Oxford; The Alan Turing Institute, UK; Bilal A. Mateen, Kings College Hospital, London, UK & The Alan Turing Institute, London, UK; Akeem Sule, Department of Psychiatry, University of Cambridge, UK; Wolfson College, University of Cambridge, UK; Essex Partnership University NHS Foundation Trust, UK; Ezinne Nwankwo, Cambridge University, UK & Harvard University, USA; Gabriela Pavarini, Department of Psychiatry, University of Oxford; Wellcome Centre for Ethics and Humanities, University of Oxford, UK; Josip Car, Centre for Population Health Sciences; WHO Collaborating Centre for Digital Health and Health Education; Health Services and Outcomes Research, LKCMedicine; Imperial College London, UK; David Crepaz-Keay, Head of Applied Learning, Mental Health Foundation, UK; Fellow, Royal Society for Public Health, UK; Jasvinder Kandola, Division of Medicine, Hammersmith Hospital Imperial College London, UK; Hannah Stewart, The University of Texas Health Science Center at Houston (UT Health) School of Public Health, Department of Health Promotion & Behavioral Sciences, Texas, USA; Eiman Kanjo, Nottingham Trent University, Smart Sensing Lab (MA220), Clifton Lane, Nottingham, UK; Sarah Ticho, Hatsumi, London, UK; April C. Foreman, American Association of Suicidology, Louisiana, USA; Emma Selby, Digital Mentality, London, UK; Stan Shepherd, Instant Access Medical, London, UK; Karen L Fortuna, Dartmouth College, Hanover, New Hampshire, USA; Emachi Eneje, Birmingham Mind, UK; Tamra Huesers, Harmony Center, Minot, North Dakota, USA; Stephen Jeffreys, Survivor Researcher Network, London, UK; Mat Rawsthorne, NIHR Biomedical Research Centre for Mental Health & Technology, University of Nottingham, UK; Gerry Craigen, Department of Psychiatry, Faculty of Medicine, University of Toronto & Associate Attending Staff Psychiatrist, Department of Psychiatry, University Health Network, Toronto General Hospital Toronto, Canada; Kristina Barger, Cogenta, UK; Neha Kumar, Georgia Institute of Technology, Atlanta, USA; Sachin Pendse, Georgia Institute of Technology, Atlanta, USA; Errin Riley, Sense About Science, London, UK; Elvira Perez Vallejos, Nottingham NIHR Biomedical Research Centre for Mental Health, UK & Institute of Mental Health, Nottingham University, UK; Mark Embrett, Dalhousie University, Canada; Ernest Okyere-Twum, Universite Paris Descartes, France & Centre for mental health research in Africa (CEMHRA); Kumar Jacob, MindWave Ventures, UK; Janak Gunatilleke, MindWave Ventures, UK; Mirantha Jayathilaka, MindWave Ventures, UK; Mariana Pinto Da Costa, Unit for Social and Community Psychiatry, WHO Collaborating Centre for Mental Health Services Development, Queen Mary University of London, London, UK; Institute of Biomedical Sciences Abel Salazar, University of Porto, Porto, Portugal; Hospital de Magalhães Lemos, Porto, Portugal; Ana Catarino, Ieso Digital Health; Ronan Cummins, Ieso Digital Health; Tom Clelford, Ieso Digital Health; James de Bathe, Ieso Digital Health; Valentin Tablan, Ieso Digital Health; Sarah Bateup, Ieso Digital Health; Andrew D Blackwell, Ieso Digital Health; Tejal Patel, Babylon; Keith Grimes, Babylon; Ed Sykes, Huma Therapuetics, UK; Pete Trainor, Vala Health; Daf Rakphetmanee, Ooca; Kanpassorn Eix Suriyasangpetch, Ooca; Annie Meharg, Kooth; Aaron Sefi, Kooth; Derek Richards, SilverCloud Health and Trinity College Dublin; Angel Enrique, SilverCloud Health and Trinity College Dublin; Jorge Palacios, SilverCloud Health and Trinity College Dublin; Antony Brown, CBTClinics; Eva Papadopoulou, Minddistrict; Charlotte Lee, Big Health; Fanny Jacq, Qare; Loïc Tse, Qare; David Plans, Huma Therapuetics, UK; Senior Lecturer in Organisational Neuroscience, University of Exeter, UK; Anika Sierk, Unmind; Heather Bolton, Unmind; Knut Schroeder, Expert Self Care; Tarek R. Besold, Alpha Health, Telefonica Innovation Alpha; Institute of Cognitive Science, University of Osnabrueck, Germany; Aleksandar Matic, Alpha Health, Telefonica Innovation Alpha; Department of Psychological and Behavioural Science, London School of Economics, London, UK; Iñaki Estella Aguerr, Alpha Health, Telefonica Innovation Alpha; Department of Electrical and Electronic Engineering, Imperial College London, London, UK; Liz Ashall-Payne, ORCHA; Rob Daly, ORCHA; Simon Leigh, ORCHA; Jo Aggarwal, Wysa; Ramakant Vempati, Wysa; Smriti Joshi, Wysa; Vinod Subramanian, Wysa; Madhura Kadaba, Wysa; Clara Falala Sechet, Owlie; Geby Chyntia Irwan, Riliv; Audrey Maximillian Herli, Riliv; Karine Chevreul, StopBlues; Anais Le Jeannic, StopBlues; Kathleen Turmaine, StopBlues; Christopher Rainbow, BeyondBlue; Megan Chor Kwan Lam, Neurum Health; Christine Hiu Man Chiu, Neurum Health; Will Allen-Mersh, Spill; Justine Roberts, Mumsnet; Sara Ray, The Mighty; Angelica Catalano, The Mighty; Jennifer Russell, TalkLife; TalkCampus; Jamie Druitt, TalkLife; TalkCampus; Boaz Gaon. Wisdo; Suzi Godson, MeeTwo; Kerstyn Comley, MeeTwo; Satu Raappana, MIELI Mental Health Finland Mental-chat & Mental Gaming, Finland; Michelle Carlson, Teen Line, USA; Andrew Parker, Papa; Ken Duckworth, National Alliance on Mental Illness, USA; Dan Gillison, National Alliance on Mental Illness, USA; Theresa Nguyen, Mental Health America, USA; Madeline Reinert, Mental Health America, USA; Victor Ugo, Mentally Aware Nigeria Initiative (MANI), Nigeria; Ifedayo Ward, Mentally Aware Nigeria Initiative (MANI), Nigeria; Chantelle Booysen, Young Leaders for the Lancet Commission on Global Mental Health and Sustainable Development; Ashley Foster-Estwick, Young Leaders for the Lancet Commission on Global Mental Health and Sustainable Development; Grace Gatera, Young Leaders for the Lancet Commission on Global Mental Health and Sustainable Development; David Karorero, Young Leaders for the Lancet Commission on Global Mental Health and Sustainable Development; Kumba Philip-Joe, Young Leaders for the Lancet Commission on Global Mental Health and Sustainable Development; Damian Juma, Young Leaders for the Lancet Commission on Global Mental Health and Sustainable Development; Claudia Sartor, Young Leaders for the Lancet Commission on Global Mental Health and Sustainable Development; Chinwendu Ukachukwu, Young Leaders for the Lancet Commission on Global Mental Health and Sustainable Development; Lian Zeitz, Young Leaders for the Lancet Commission on Global Mental Health and Sustainable Development; Alex Fine, Qntfy, USA; Merlyn Holkar, Money & Mental Health Policy Institute, UK; Conor D'Arcy, Money & Mental Health Policy Institute, UK; Katie Alpin, Money & Mental Health Policy Institute, UK; Jo Kerr, Turn2Us, UK; Lee Healey, IncomeMax, UK; Olly Betts, Tully and OpenWrks Group, the team behind Tully; Andrea Severino, Healthy Virtuoso, Italy; Will Van Der Hart, The Mind and Soul Foundation, UK; Danielle Smalls, The TellFinder Alliance, USA; Chris Dickson, The TellFinder Alliance, USA; Andrew Stroz, The TellFinder Alliance, USA; Sebastian Vollmer, Warwick University, Warwick, UK & The Alan Turing Institute, London, UK; Hoang D. Nguyen, School of Computing Science, University of Glasgow, Singapore; Daniel Albert Rosello, Nottingham Trent University, Nottingham, UK; Valentino Megale, Softcare Studios, Rome, Italy; Jan D. Smeddinck, Open Lab, Newcastle University, Newcastle upon Tyne, UK; Rosanna Bellini, Open Lab, Newcastle University, Newcastle upon Tyne, UK; Craig A. DeLarge, The Digital Mental Health Project, Wise Working, California, USA; Shivani Patel, South London and Maudsley NHS Trust, London, UK; Jerome Uriko-kang, Global Mental Health Peer Network, Ghana, Africa; Tunde Olatunji, Lyrical Kombat, London, UK; Vanessa Lalo, Liberal Clinical Psychologist, Paris, France; Robert Walker, Department of Mental Health Office of Recovery and Empowerment, Massachusetts Department of Mental Health, USA; Ann John, Population Data Science, National Centre for Mental Health, Swansea University, Swansea, Wales, UK; Diana Rayes, The Johns Hopkins Bloomberg School of Public Health, Baltimore, USA; Marwa Elnahass, Newcastle University, UK; Karen Elliott, Newcastle University, UK; Lil Tonmyr, Family Violence, Mental Health & Suicide Surveillance Team, Behaviours, Environments and Lifespan Division, Centre for Surveillance and Applied Research, Public Health Agency of Canada; Andrew MacKenzie, Centre for Surveillance and Applied Research, Public Health Agency of Canada; Michael L. Birnbaum, The Zucker Hillside Hospital, Psychiatry Research, Northwell Health, New York, USA; Eric D. Caine, University of Rochester Medical Center, New York, USA; John Pestian, Cincinnati Children's Hospital Medical Center, University of Cincinnati; Oak Ridge National Laboratory/VA-MPV Champion program, Cincinnati, USA; Dan Jacobson, Oak Ridge National Laboratory/VA-MPV Champion program, Cincinnati, USA; Mike Sorter, Cincinnati Children's Hospital Medical Center, University of Cincinnati, Cincinnati, USA; Tracy Glauser, Cincinnati Children's Hospital Medical Center, University of Cincinnati, Cincinnati, USA; Michael Meaney, Translational Neuroscience programme, Singapore Institute for Clinical Sciences, Singapore & McGill University, Quebec, Canada; Vincent M. B. Silenzio, Rutgers School of Public Health, Rutgers University, New Brunswick and Newark, New Jersey, USA; Jenny Edwards, London, UK; Ricardo Araya, Centre for Global Mental Health, King's College London, UK; Chris Fitch, Personal Finance Research Centre, University of Bristol, UK; Jamie Evans, Personal Finance Research Centre, University of Bristol, UK; Kevin Telford, University of Edinburgh, Scotland, UK; Peggy Loo, Legal & General Group Plc, UK; Andrea Stevenson, Independent Consultant, London, UK; Tatyana Marsh, Open Banking Excellence, UK; Helen Child, Open Banking Excellence, UK; Roger S. McIntyre, Mood Disorders Psychopharmacology Unit, University Health Network, Toronto, Canada & Institute of Medical Science, University of Toronto, Toronto, Canada & Department of Pharmacology, University of Toronto, Toronto, Canada & Department of Psychiatry, University of Toronto, Toronto, Canada & Brain and Cognition Discovery Foundation, Toronto, Canada; Henrietta Bowden-Jones, National Centre for Behavioural Addictions, UK (National Problem Gambling Clinic + National Centre for Gaming Disorders) & Medical Women's Federation & Royal Society of Medicine & Royal College of Psychiatrists & University College London, UK; John Torous, Digital Psychiatry Division, Department of Psychiatry, Beth Israel Deaconess Medical Center, Harvard Medical School, Harvard University, Massachusetts, USA; Thomas R. Insel, Humanest Care, USA.

## ORCID IDs

Becky Inkster: 0000-0003-1201-455X; Anne-Claire C. Stona: 0000-0003-0350-0345; Philip Resnik: 0000-0002-6130-8602; Terry Hanley: 0000-0001-5861-9170; David Crepaz-Keay: 0000-0003-3845-4721; Neha Kumar: 0000-0002-7014-5585; Ann John: 0000-0002-5657-6995; Mariana Pinto Da Costa: 0000-0002-5966-5723; Mark Embrett: 0000-0002-3969-0219; Munmun De Choudhury: 0000-0002-8939-264X; Mat Rawsthorne: 0000-0002-7481-693X; Vincent M. B. Silenzio: 0000-0003-1408-7955; Aleksandar Matic (Alpha Health): 0000-0002-8752-4098; Sebastian Vollmer: 0000-0002-9025-0753; Jo Robinson: 0000-0001-5652-918X; Pete Trainor (Vala Health): 0000-0002-7778-3934; Karen L Fortuna: 0000-0003-0343-2346; Pattie Pramila Gonsalves: 0000-0003-3780-4523; Smriti Joshi: 0000-0001-7446-2804; Hannah Stewart: 0000-0003-2536-9405; Tarek R. Besold (Alpha Health): 0000-0002-8002-0049; Mirantha Jayathilaka: 0000-0002-2462-4833; Gabriela Pavarini: 0000-0001-5574-4021; Sarah Bateup: 0000-0003-3926-0021; Iñaki Estella Aguerri: 0000-0001-5110-6858; Janak Gunatilleke: 0000-0003-1474-5735; Hoang D. Nguyen: 0000-0003-2541-3269; Ricardo Araya: 0000-0002-0420-5148; Karen Machin: 0000-0002-0374-4238; Glenn Melvin: 0000-0002-6958-3908; John A. Naslund: 0000-0001-6777-0104; Stephen Jeffreys: 0000-0002-5088-9309; John Torous: 0000-0002-5362-7937; Thomas R. Insel: 0000-0001-5031-0160; Bo Wang: 0000-0002-3412-3768; Abdullahi Abubakar Kawu: 0000-0003-2531-9539; Lil Tonmyr: 0000-0002-8722-7616; Clara Falala-Séchet (Owlie): 0000-0003-3331-5255; Maria Liakata: 0000-0001-5765-0416; Simon Leigh: 0000-0002-6843-6447; Liz Ashall-Payne: 0000-0001-7325-195X; Karen Elliott: 0000-0002-2455-0475; Sarah Ticho: 0000-0002-5585-0497; Bilal A. Mateen: 0000-0003-4423-6472; David Plans: 0000-0002-0476-3342; Rosanna Bellini: 0000-0002-2223-2801; Tejal Patel: 0000-0002-7356-7054; Derek Richards: 0000-0003-0871-4078; Jorge Palacios: 0000-0002-2103-5507; Angel Enrique: 0000-0003-0585-4008; Niranjan Bidargaddi: 0000-0003-2868-9260; Valentino Megale: 0000-0003-2150-696X; Elvira Perez Vallejos: 0000-0002-0258-9440; Aaron Sefi: 0000-0002-0776-3858.

## References

[1] World Health Organisation. Global Burden of Mental Disorders and the Need for a Comprehensive, Coordinated Response From Health and Social Sectors at the Country Level. (2011). Available online at: https://apps.who.int/gb/ebwha/pdf_files/EB130/B130_9-en.pdf (accessed May 17, 2020).

[2] Yip PS Cheung YT Chau PH Law YW. The impact of epidemic outbreak: the case of severe acute respiratory syndrome (SARS) and suicide among older adults in Hong Kong. Crisis. (2010) 31:86–92. 10.1027/0227-5910/a000015 20418214

[3] Sprang G Silman M. Posttraumatic stress disorder in parents and youth after health-related disasters. Disaster Med Public Health Prep. (2013) 7:105–10. 10.1017/dmp.2013.22 24618142

[4] Granados JAT Roux AVD. Life and death during the great depression. Proc Natl Acad Sci USA. (2009) 106:17290–5. 10.1073/pnas.0904491106 19805076 PMC2765209

[5] Gunnell D Chang SS. Economic recession, unemployment, and suicide. In: O'Connor RC, Pirkis J, editors. The International Handbook of Suicide Prevention. New Jersey; New York, NY: John Wiley & Sons, Ltd. (2016). p. 284–300. 10.1002/9781118903223.ch16

[6] Lai J Ma S Wang Y Cai Z Hu J Wei N. Factors associated with mental health outcomes among health care workers exposed to coronavirus disease 2019. JAMA Netw Open. (2020) 3:e203976. 10.1001/jamanetworkopen.2020.3976 32202646 PMC7090843

[7] Tan BYQ Chew NWS Lee GKH Jing M Goh Y Yeo LLL. Psychological impact of the COVID-19 pandemic on health care workers in Singapore. Ann Intern Med. (2020) 17:M20-1083. 10.7326/M20-1083 32251513 PMC7143149

[8] Greenberg N Docherty M Gnanapragasam S Wessely S. Managing mental health challenges faced by healthcare workers during covid-19 pandemic. BMJ. (2020) 368:m1211. 10.1136/bmj.m1211 32217624

[9] Impact of COVID-19 on Black Asian and Minority Ethnic (BAME) Staff in Mental Healthcare Settings|Assessment and Management of Risk. (2020). Available online at: https://www.rcpsych.ac.uk/news-and-features/latest-news/detail/2020/06/11/more-support-needed-for-bame-psychiatrists-during-the-pandemic-according-to-rcpsych-survey (accessed May 17, 2020).

[10] Domestic Abuse Killings ‘More Than Double' Amid Covid-19 Lockdown. (2020). Available online at: https://www.theguardian.com/society/2020/apr/15/domestic-abuse-killings-more-than-double-amid-covid-19-lockdown (accessed May 17, 2020).

[11] Child Abuse: L'express. Violences intrafamiliales: lors du confinement, les appels au 119 ont presque doublé. (2020). Available online at: https://www.leparisien.fr/faits-divers/violences-intrafamiliales-les-appels-au-119-ont-presque-double-22-04-2020-8304171.php (accessed May 17, 2020).

[12] Kempson E Poppe C Standard Life Foundation. Coronavirus Financial Impact Tracker. (2020). Available online at: https://www.standardlifefoundation.org.uk/__data/assets/pdf_file/0030/57486/COVID-19-Financial-Impact-Tracker-April-2020-FINAL.pdf (accessed May 17, 2020).

[13] Keeter S. People Financially Affected by COVID-19 Outbreak Are Experiencing More Psychological Distress Than Others. Pew Research Center (2020). Available online at: https://www.pewresearch.org/fact-tank/2020/03/30/people-financially-affected-by-covid-19-outbreak-are-experiencing-more-psychological-distress-than-others/ (accessed May 17, 2020).

[14] Richardson T Elliott P Roberts R. The relationship between personal unsecured debt and mental and physical health: a systematic review and meta-analysis. Clin Psychol Rev. (2013) 33:1148–62. 10.1016/j.cpr.2013.08.009 24121465

[15] Inkster B Loo P Mateen B Stevenson A. Improving insights into health care with data linkage to financial technology. Lancet Digit Health. (2019) 1:110–2. 10.1016/S2589-7500(19)30061-5 33323259

[16] Skatova A Shiells K Boyd A. Attitudes toward transactional data donation and linkage in a longitudinal population study: evidence from the Avon Longitudinal Study of Parents and Children. Wellcome Open Res. (2019) 4:192. 10.12688/wellcomeopenres.15557.1 32685696 PMC7341003

[17] Frankham C Richardson T Maguire N. Psychological factors associated with financial hardship and mental health: a systematic review. Clin Psychol Rev. (2020) 77:101832. 10.1016/j.cpr.2020.101832 32088498

[18] Holmes EA O'Connor RC Perry VH Tracey I Wessely S Arseneault L. Multidisciplinary research priorities for the COVID-19 pandemic: a call for action for mental health science. Lancet Psychiatry. (2020) 7:547–60. 10.1016/S2215-0366(20)30168-1 32304649 PMC7159850

