# Supplementary material for: Early Warning Signs of a Mental Health Tsunami: A Coordinated Response to Gather Initial Data Insights From Multiple Digital Services Providers
Source: Front Digit Health. 2021 Feb 10;2:578902. doi: 10.3389/fdgth.2020.578902 (PMC8521957; doi:10.3389/fdgth.2020.578902)
Supplement: Supplementary file 1 [file Table_1.docx]

**Table 1:**  A list of digital services providers who shared data insights for our project

| **Mental health and wellness services providers** | |
| --- | --- |
| **Patient to clinician communication tools** | |
| Ieso Digital Health | **UK**; biggest UK provider of online CBT; serving approximately 20 million people; commissioned in 74 NHS clinical commissioning group areas in England and 14 health boards in Scotland; <https://www.iesohealth.com/en-gb/> |
| Babylon | **International**; Findings reported here comes from UK NHS arm with 78,000 registered patients via Babylon GPathand; <https://www.babylonhealth.com/> |
| Vala Health | **UK** (c5000 members), **Australia** (c1000 members), **Israel** (data not stored) |
| Ooca | **Thailand;** telemental health platform with 72,000 users - face-to-face video counseling service for individual user and mental health support service as a corporate welfare for employee; [www.ooca.co](http://www.ooca.co) |
| **Digitally-enabled treatments** | |
| Kooth | **UK**; One of the largest providers of digital mental health services to the NHS in the UK, enabling 1-1 counselling with professionals, alongside peer support and self help tools. Kooth is for children and young people in 135 NHS clinical Commissioning Group areas, and is available to over 5 million children and young people seeing 3,800 unique logins every day (Kooth.com). Qwell is for adults is available to 500,000 people in commissioned areas to cohorts including teachers, parents, people who have suffered/who are suffering from domestic violence (Qwell.io) |
| Silver Cloud Health | **Europe & North America**; https://www.silvercloudhealth.com/uk |
| CBTClinics | **UK**; Network of 4,500 accredited clinicians and 25 Internal clinicians. Treating 25,000+ patients per year. [www.cbtclinics.co.uk](http://www.cbtclinics.co.uk); 2005 |
| Minddistrict | **Europe & North America:** over 200.000 users per year; digital platform and mobile app with guided interventions and self-help training for mental and behavioural health; <https://www.minddistrict.com/> |
| **Self-managed care solutions** | |
| Big Health | **US:** 1.4m people covered as part of COVID-19 offer. **UK:** 3.3m people covered with the majority being health and social care staff (3m) as part of a partnership with NHS England, National Care Forum (England) and NHS Scotland (Scotland); [www.bighealth.com](http://www.bighealth.com); Our COVID-19 offer was launched 20th March with NHS England, the 23rd April with social care, and due to be launched 30th April with NHS Scotland. |
| Qare | **France/UK**; a telemedicine platform for mental health consultations with psychiatrists, among other medical specialties (GPs, etc). 2017.  Qare also developed an app ([Mon Sherpa)](https://monsherpa.io/) to support people with mental health problems in between consultations. 2019. |
| Biobeats | 54.55% of users are logged from UTC+0, while 43.31% from UTC+1. The rest 2.14% of times the user are logged from UTC outside of Europe; for this product 3,000 users; [www.biobeats.com](http://www.biobeats.com/); 2019 |
| Unmind | **UK**; Predominantly UK; analysed circa 20,000 users. workplace mental health platform; working-age adults, employed in a variety of sectors; [www.unmind.com](http://www.unmind.com/); 2016 |
| Expert Self Care (distrACT app) | **UK** (majority); suicide and self-harm app; 25,000 downloads; <https://www.expertselfcare.com/health-apps/distract/>; 2017. |
| Alpha Health | **US, UK, Spain**; we report results from a limited trial including 2500 users that was launched on the 10th of February 2020.  <https://evermind.health/> |
| Neurum Health | **Vietnam, Singapore, Hong Kong, Mainland China;** digital behavioural health platform, primarily workforce-based, across sectors; N=1050 sample reported here; <https://www.neurumhealth.com/>; 2018. |
| **Other (Clinical)** | |
| Improving Access to Psychological Therapies (IAPT) NHS London Clinical Leads | **Whole of England**; Data was sourced for the London region where IAPT services cover a population of circa 9 million people; 2008 |
| ORCHA | **World’s leading** health app evaluation and advisor organisation. With over 4,000 health-apps reviewed to date and around 50,000 pageviews/month. |
| Public Mental Health Services South Australia, Clinical Leads | **Australia**; Data was provided by clinical leads at two public mental health services in South Australia: 1) IAPT, a service attached to Flinders Medical Centre that provides phone based CBT to the population of the Southern Adelaide region and 2) Regional Mental health services, severe mental illness case management to populations of regional South Australia. |
| **Apps (e.g., chatbots/text-based therapists etc)** | |
| Wysa | **Global** (general trend): US, 30-40%; UK, Canada, ANZ, 10-15%; India, 15-20%; South East Asia (Indonesia, Philippines, Malaysia, Singapore), 15-20%; Rest of the world, 15-20%; 1.9 million installs / users now and adding ~80-100K per month; <https://www.wysa.io>; 2017 |
| Owlie | **French speaking users globally**; 16,000 users; chatbot-based emotional support for coping with psychological difficulties.  2018. <https://m.me/owlielechatbot>. |
| Riliv | **Indonesia;** currently has 200,000 users, with 70% are female in their 18-24 years old, living in Indonesia's big cities such as Jakarta and Surabaya. |
| StopBlues | **France;** 270,000 free access, 11,200 have an account; [https://www.stopblues.fr](https://www.stopblues.fr/); 2018 |
| BeyondNow | **Australia**; 100,000+; suicide prevention app; <https://www.beyondblue.org.au/get-support/beyondnow-suicide-safety-planning>; 2016 |
| Spill | **UK, Europe;** Provider of video and message-based therapy to employees of organisations; 1,000+ users <https://www.spill.chat> |
| **Forums, support networks, and digital communities** | |
| Mumsnet | **UK**; Mumsnet is the UK’s largest network for parents, with around 10 million unique visitors per month and ~100 million page views; [www.mumsnet.com](http://www.mumsnet.com/) |
| The Mighty | **Global**; 3 million registered community users, 5 million monthly visitors;  [www.themighty.com](http://www.themighty.com/); Started in 2014; iOS app launch in 2018; Android app launched in 2019 |
| TalkLife & TalkCampus | **Global** (125 countries); TalkLife: 2.1 millions registered members and 29.5 million content shared monthly  **Global**; TalkCampus: 45 registered universities and 310,000 university students covered. |
| Wisdo | **US, UK, Canada, New Zealand, Australia;** peer-peer support community; 1.5 million, 26 million conversations, 27 million "Life Events" submitted, ~100 million content impressions; 19 sessions on av. for people looking for support; 35 sessions on av. for people giving support ("Helpers"); <https://wisdo.com/>; 2018 |
| MeeTwo | **UK**; 25K young people aged 11-25 throughout UK; 100% pre-moderated by humans peer support social media app; <https://www.meetwo.co.uk>; 2017 |
| MIELI Mental Health Finland: Mental-chat &  Mental Gaming | **Finland,** 1 to1 chat to 12 to 29 years old young people. Anonymous, free of charge. 21,000 conversations yearly, over 115,000 dropped conversation requests in 2019 due to long queues. At the moment over 1100 social work professionals and trained volunteers are working on the chat. [www.sekasin.fi](http://www.sekasin.fi)  **Finland,** As a part of Mental-chat “family”, we run a Discord server and a community that aims to promote the good sides to e-gaming and to give a place and a voice especially to young men, who are in danger of being left out of the society, education, work and relationships . At the moment the server is biggest and busiest in Finland, with 13 064 members. |
| Teen Line | **USA**; youth peer hotline & services; 20,000+; [www.teenlineonline.org](http://www.teenlineonline.org) |
| Papa | **USA** (17 states)**;** virtual companionship between elderly and young people; Active membership has doubled since COVID; [www.joinpapa.com](http://www.joinpapa.com/); 2016 |
| Orygen | **Australia**; in the past year, 250 youth partners, 1640 training participants, 37 research projects, 17 clinical trials; https://www.orygen.org.au |
| Digital Peer Support | **USA (24 different states), Australia, Europe, Asia**; [www.digitalpeersupport.org](http://www.digitalpeersupport.org); March 10, 2020 |
| **Digital campaigns and charities** | |
| National Alliance on Mental Illness (NAMI) | Users from **237 countries** visited NAMI.org, (81% from the U.S.) (Top 5 countries: US, UK, Canada, Australia and India); In 2019, more than 12 million users from 237 countries visited; NAMI.org; 1979 |
| Mental Health America | **USA** (90%) **International** (10%); provide anonymous and free validated mental health screens to users online for depression, anxiety, psychosis, bipolar, ptsd, addiction, eating disorder, and a parent and youth screen; Average of 1 million users to take a screen every year; demographics match census, trend younger; [www.mhascreening.org](http://www.mhascreening.org) |
| Mental Health Foundation | **UK;** The Mental Health Foundation, a UK NGO, is regularly polling UK citizens on a range of Coronavirus issues pertaining to mental health.  Survey polling UK adults, aged 18+ sample sizes n1=2126, n2=2221, n3=4246. First survey 17-18 March, repeated 2-3 April with some questions added, third wave 24-26 April sample size doubled.  www.mentalhealth.org.uk |
| Sangath | **India;** www.sangath.in;1996 |
| Its OK To Talk | **India**; a public engagement program by Indian NGO, Sangath; Reached 7000+ directly through over 75 community events and workshops; worked with 280 volunteers, trained 50 youth advocates in mental health leadership and reached more than 2.5 million users on social media; 2016. |
| Mentally Aware Nigeria Initiative (MANI) | **Nigeria**; Nigeria's largest youth-run and youth-focused mental health organization; Active online presence: 31.9k followers / 37.3k tweets; [mentallyaware.org](http://mentallyaware.org/) & <https://twitter.com/MentallyAwareNG>; 2016 |
| Young Leaders for the Lancet Commission on Global Mental Health and Sustainable Development | **Global**; Group of mental health advocates, largely from low and middle income countries, who support young people in their role as agents of change for global mental health (www.globalmentalhealthcommission.org/youth-campaign). They lead a global campaign titled “My Mind Our Humanity”: [www.instagram.com/mymindourhumanity](http://www.instagram.com/mymindourhumanity) |
| Money and Mental Health Policy Institute | **UK**; an interest based sample of 568 people (not representative of the wider 5,000 people making up their research community) with lived experience of mental health problems; https://www.moneyandmentalhealth.org/coronavirus-policy/ |
| **Other digital health insight providers** | |
| Qntfy | **USA** (majority); analytics provider, results based on 25,046 healthcare professionals, and 10,000 community controls. <https://qntfy.com/cap>; Qntfy in 2015; Cohort Analytics Platform in 2019. |
| Practicing NHS clinician | **UK**; Jasvinder Kandola, North West London and Dorset NHS Trust. |
| Google trend analytics | **UK**; analysed by Hannah Stewart (using freely available Google tools; independent insights - this work was not carried out by Google) |
| Consultant NHS nurse | **UK**; collecting by Emma Selby; Anonymous user and staff feedback/messages. |
| **Broader services providers and other relevant digital data sources (e.g., financial, fitness, religious, dark web and darknet markets)** | |
| **Financial services providers** | |
| Turn2us | **All of the UK**; Last year, 2.3 million benefit calculations were conducted on our site, by 1.2 million households. Since 16th March, over 800,000 benefits calculations have been completed. We also provide direct grants to people in financial need. Since Covid-19, we had 4,500 applications for a crisis grant in the first two days.<https://www.turn2us.org.uk/> Started providing individual grants in 1897 and our digital tools, such as the benefits calculator, began in 2013. |
| IncomeMax | **UK**; Personal money advice service; http://www.incomemax.co.uk |
| Anonymous financial services provider | A large anonymous financial services provider |
| Tully and OpenWrks Group | **UK** (primarily England); Fintech; 14,000+ users; <https://www.tully.co.uk/> |
| **Other digital data sources** | |
| Healthy Virtuoso | **Italy** (mainly); 130.000 active user who on average connect 40 times per month; [www.healthyvirtuoso.com](http://www.healthyvirtuoso.com/); 2019 |
| The Mind and Soul Foundation | **UK/USA**; ~ 500,000 page requests on web site; <https://www.mindandsoulfoundation.org>; 2005 |
| The TellFinder Alliance | **USA**; <https://tellfinder.com/>; Dark web qualitative insights and darknet market analysis insights |
