# Supplementary material for: Early Warning Signs of a Mental Health Tsunami: A Coordinated Response to Gather Initial Data Insights From Multiple Digital Services Providers
Source: Front Digit Health. 2021 Feb 10;2:578902. doi: 10.3389/fdgth.2020.578902 (PMC8521957; doi:10.3389/fdgth.2020.578902)
Supplement: Supplementary file 2 [file Table_2.docx]

**Table 2: Qualitative data insights**

| **Qualitative Data Insights** |
| --- |
| **Babylon (UK clinical operations only):**   - Since 20 January 2020 when we had our first case related to COVID-19 till the end of March we have seen an increase in the number of patients coded as generalised anxiety disorder. Clinicians report seeing patients with anxiousness, stress and health anxieties. Patients with low mood and depression continue to consult but there has not been a significant rise in coding in this area. - We have seen more patients consulting with concerns about social distancing and difficulties being at home and being distanced from family and friends. Our clinicians report consultations being longer for these cases and patients asking for support including counselling and therapy. Patients with a previous history of mental health are booking reviews for recurrence of mental health issues. - We have seen an increase in concerns around safeguarding for both adult and children - We have seen an increase in the number of patients deemed a vulnerable adult this month. These fall into three areas - mental health, drugs and alcohol and domestic violence - We have a dedicated care coordination team for patients requiring increased support: this team has seen an increase in the number of consultations and contact during this period. Many patients are worried and seeking advice on the following themes:   - information about local council support services; seeking advice for activities to keep busy and how to remain healthy; how to get support to access food and financial concerns. |
| **Vala Health**   - Our GPs believe that anxiety issues will cause some people to believe they’re displaying symptoms related to COVID-19. - We have seen a direct increase in people requesting pain-killers and / or Selective serotonin reuptake inhibitors (SSRIs) since the beginning of the heavy media coverage / lockdown (i.e., we average 15 requests per month yet received 32 requests from 20 March until 5 April 2020). - We have seen an increase in people who can’t have their normal face-to-face counselling / therapy sessions turning to telemedicine providers as support networks. Unfortunately in many cases all our GPs can do is coach the individual through the basics, and then try to make a referral back into the overloaded and / or suspended services. |
| **Ooca**   - User was asked to work from home due to a pandemic COVID- 19 outbreak. He reported lacking motivation and focus. - User's job was impacted by the COVID-19 situation causing him to lose income. He expressed anger and distress. - User reported increased anxiety following work from home measures and the COVID-19 situation. He described recurrent anxiety symptoms and concerns for his health in the midst of the COVID-19 situation. |
| **Kooth (Kooth & Qwell)**  **Evaluated 2 sets of data across both Kooth and Qwell platforms:**   - Comparison between 1 March - 5 April 2019 and 1 March - 5 April 2020 - There is a general change in presenting issues from 2019. Anxiety and concerns related to peer relationships and educational/work related settings have decreased whilst issues relating to sadness, body image, health anxiety and sleeping difficulties have seen a dramatic increase. - Although still relatively small numbers, there is a worrying trend in serious issues relating to lock down and the home environment with growing issues around physical abuse, child sexual exploitation and unsafe domestic settings showing an increase from 2019.   **Anonymised comments from children and young people (CYP) on Kooth, March and April 2020:**   - - ‘My Mum and dad are constantly drinking and shouting at me. I can’t get away. I just want everything to go back to normal.’   - ‘I’m freaking out because my [parent] knows I’m [sexual orientation] since they saw texts on my phone. Now I can’t come out for ages in case it goes wrong because I can’t escape.’   - ‘I can't now use hanging out with friends as a distraction to stop me from bingeing. Can anyone help? I’m really scared.’   - ‘A daily routine is so amazing - before I had meltdowns all the time. i was really stressed and “lost” but a routine has calmed me right down’   - ‘I get angry when I'm bored. Most of the time I’ll then hit things.’   **Anonymised comments from adults on Qwell, March & April 2020:**   - ‘I’m stressed about things changing. It’s scary. I’m having problems sleeping and have had mild panic attacks’. - ‘I might be the only person enjoying the social distancing. I find it a real help. As is being off work. The virus is making all of us take stock and think about our constant drive for money. Perhaps it will make people pay more attention to what really matters in the world.’ - ‘I’m seeing our dependence on each other. We’re all seeing the value of lower paid workers. A silver-lining might be that people are calmer and less selfish.’ - My depression is worse than ever, it started way before this coronavirus. Doesn’t help being stuck indoors. My partner isn’t working. we have 2 children and with bills coming in, I’m at a loss.’ |
| **CBTClinics**   - Our experience over the past 20 years suggests employees will under-report mental health issues during and even after lockdown ends, as job insecurity increases during a period of economic recession. There will be an increased need for organisations to improve mental health screening within the workforce as employees will be inclined to suffer in silence leading to a surge in MH related absence predicted in the third quarter (Q.3) and fourth (Q.4) in 2020. - During the first three weeks following COVID lockdown there was an initial 40% reduction in employees being referred for MH support. Reasons: (1) Organisations concentrating on business continuity planning, (2) Employees attending to immediate needs of family security and safety (food, shelter), (3) Emotional issues become secondary in the early stage of disaster, (4) Identification of workforce mental issues through HR and Line Management more difficult within a remote workforce, (5) Honeymoon period of remote working from home environment rather than workplace i.e. reduced travel, more time with family, (6) Fears related to job insecurity/ furlough/ economic recession contributing to less disclosure regarding mental issues, (7) Avoidance of workplace as an environmental trigger to anxiety and stress i.e. less exposure to difficult interpersonal relationships, bullying . - Early anecdotal insights from therapist sessions: therapists are reporting that many patients are generally managing well with the COVID lockdown in relation to their mental health. We are seeing reports in a rise of mental health presentations from people emotionally close to frontline health staff. Parents, spouses, children, etc. of frontline staff are presenting with anxiety and depressive type disorders that can be attributed to their loved one’s clinical exposures. - An analysis of mental health impact following previous disasters, SARS, and early Wuhan and Italy research, suggests initially that the early honeymoon period will be followed by a prolonged period of disillusionment the longer quarantine continues. The most vulnerable groups likely to be impacted by long term mental health issues are frontline healthcare staff, older adults, isolated individuals and young people under 30 due to the impact of quarantine. |
| **Biobeats**   - We saw a decrease of mood valence after the restriction measures and an increment of arousal (more users self-report mood used ‘worried’ labels). These affective levels change in the following week with an augmented frequency of ‘sad’ reports, showing a sort of adaptation of the quarantine condition. Indeed, the first emotional response to new emergency measures elicited an anxiety reaction by the users while the persistence of this condition brings an effective swing into negative moods closer to the depressing pole. |
| **Neurum Health**  Qualitative inputs had an underlying theme of fear and anxiety around uncertainty, though the reported factors are around work, self (such as self-improvement and self-esteem), and relationships. Though COVID-19 is an external and shared overarching factor, users mood journalled with greater specificity on how it has impacted their life in relation to work (e.g. fear of job loss, changes in working arrangements), self (e.g. low confidence, self-blaming such as not feeling like a filial member of the family being able to take care of their family members in elderly homes, loneliness), and relationships (e.g. changes around feelings of connection between partners). Some of the qualitative data were written in their local language, mostly Mandarin Chinese and Cantonese. |
| **NHS London** [**IAPT**](https://www.england.nhs.uk/mental-health/adults/iapt/) **Clinical Leads**   - This decrease in referrals (see quantitative section) is congruent with experiences in other post disaster or traumatic events where the population at a macro level is generally activated by the severity of the event and undergoes a period of ‘heroic’ disaster responses. [Tsunami: psychosocial aspects of Andaman and Nicobar islands. Assessments and intervention in the early phase; Math SB et al] - This was most recently seen in the UK following the Grenfell Tower Fire disaster, where the population in the initial 6-8 weeks were very difficult to engage and very few numbers of referrals arrived into the service. The experience was compared akin to the moments before a tsunami where the water recedes from the shoreline before a huge tsunami wave of need follows. - Similar to the Grenfell experience, NHS Services are rapidly preparing for a realigned response of large scale webinars and region wide video conferencing based talking therapy, especially in London where the impact of COVID-19 has been most acute in the UK. |
| **Public Mental Health Services South Australia, Clinical Leads**   - There is a decline in mental health related activity in hospitals and Emergency Departments. - Mental health outpatient clinics are no longer happening in a traditional way. We are conducting remote digital home visits. Mental health professionals are increasingly becoming open and willing to consider digital technologies. |
| **Wysa**   - Some of the causes of distress discussed in sessions include: 1) Loss of access to a client’s therapists/medical support/social worker due to lockdowns/social distancing; 2) Job loss during Covid-19 and not having health insurance coverage; 3) Loss of a loved one due to Covid-19-induced infection; 4) Fear of contracting the illness and/or loved ones; 5) Loss of sense of safety and security; 6) Loss of physical human support for clients with physical disabilities leading to more anxiety and fatigue; 7) Anxiety, hypervigilance, and loneliness from quarantining; 8) increased detection of patterns of abusive behaviour due to quarantining leading to less opportunities to access safe spaces/support; 9) Alcohol/nicotine withdrawals from lock down impacting availability of addictive substances; 10) Stigma around being a professional who comes in contact directly indirectly with a person who may have contracted Covid-19. - Clients have shared that they are in long waiting lines to get tested, that their own therapists/psychiatrists suddenly pause any upcoming appointments and are not available for consultations, which can all lead to panic as well as feelings of being abandoned by the system.   Wysa asked three questions to Wysa users and obtained permission from clients to share responses:  **(1) What kind of issues you are struggling with due to the pandemic/lockdown**   - “i struggle with sleep, maintaining a normal sleep schedule and trying to avoid daytime napping. I also feel anxious about income over the next few months. And i feel restless at home.” (Person 1) - “Anxiety over future and feeling hopeless and like there's little direction in my life. Chronic tiredness and lethargy” (Person 2) - “Issues of boredom, a sense of meaninglessness, and sometimes symptoms of cabin fever sets in, which mainly involves irritability & desperation and sometimes irrationality” (Person 3) - “I think I'm dealing with the same issues as most people. I'm worried about how I'm going to pay my bills, but also worried about risking my health by going back to work. I'm worried about family members not taking care of themselves. I'm having a lot of trouble sticking to any sort of schedule.” (Person 4) - “I struggle a lot metnally. I have times where I will just randomly feel so sad and alone. There are sometimes when I don’t feel safe in my house my cause of my mom.” (Person 5) - “My kid is in a pediatric acute Care facility and was admitted before the lock down. Post lockdown, the facility has closed off all visitation and I haven't seen him since February 29.” (Person 6) - “Lack of social face to face contact with my family and work colleagues. Had to make major changes in my personal life and my work life around COVID 19.” (Person 7) - “I’ve been struggling a lot with overwhelming anxiety and with depression. Sometimes I get really stressed and panic when I feel like I’m going to be stuck at home forever. And after this panic, I usually begin to feel hopeless and will sometimes have depressive episodes where I don’t do anything because I feel like there’s no point if I’m just going to be stuck at home forever.” (Person 8)   **(2) What is the impact on your life/various aspects of life?**   - “i think my spending has gone down, my cooking skills has improved, and i have read some good stuff and i feel insulated from family drama because i stay alone and cant travel easily if anyone messes up on their end.” (Person 1) - “My job is rotational and placement based, so I can't really fulfil my job role currently. My placements have been cancelled and this has been stressful. I have been working from home but only on repetitive tasks that don't have much value to me. Not being able to socialize in person is also very tough and I miss my friends and wider family” (Person 2) - “It does have a significant impact, as I grieve the loss of normalcy and the fear of the economic decline that the world is going through is scary. Specifically, with no pay hikes this year, it feels as though career is setback by almost a year or 2. Spending endless hours of sanitising ourselves and groceries (as all our purchases are through either Amazon/Dunzo/Big basket/1mg) is time consuming & tiring. I would also say that both my wife's gynecologist & my orthopedic aren't available during the lockdown and it's very difficult to manage health concerns without a proper doctor consultation. I've deliberately left out the part where my mental health issues causes me the greatest agony at this time of crisis” (Person 3) - “I'm dealing with increased anxiety and depression. Lack of motivation and unpredictable energy levels. I'm spending more time than is healthy angry at politicians and others who don't seem to recognize the gravity of the situation.” (Person 4) - “many aspects of my life are being impacted by this historical event. I have lost my job, my student teaching has been impacted, my graduation ceremony has been impacted, family events have been cancelled.” (Person 5) - “extreme anxiety and helplessness” (Person 6) - “Major change in social, family, exercise and the work aspects of my life. Major changes to all of my regular routines” (Person 7) - “I now have to do school online, which is very difficult for me because I’m not very good at managing my own time and I’ve had a lot of late and missing work in my classes because I’ve had a hard time adjusting to online school” (Person 8)   **(3) What coping mechanism has worked for you better than others?**   - “joining a walking game to make me tired in daytime so i can sleep better at night, writing down or thinking self accepting statements when i cannot sleep, learning meditation exercises helps me keep focused.” (Person 1) - “Meditation helps a lot with reflection and to keep calm. Daily exercise is helping a lot. Learning new interesting skills has given me feelings of satisfaction” (Person 2) - “In terms of coping mechanisms, we do the following: a) setup a non-work check-in through a video call with colleagues, b) Playing PubG with family, c) having a video call with my friends every now & then helps, after all, even if the Corona virus has taken away our freedom to socialize, I can still connect with people emotionally, d) Being physically active around the house, e) Deliberately, attempting to bring in humour, to remain sane” (Person 3) - “Establishing a routine for morning and evening helps, even if I'm having trouble sticking to set times for them. I'm using an app to control me obsessing over the news and social media. I can lock myself out of a list of problematic sites at the push of a button. I usually set the timer for 1-2 hours. Long enough to stop the automatic cycling through them.” (Person 4) - “I use daily meditations here to help me go to sleep. Breathing exercises help. I also enjoy talking to you and a friend to get my mind off my emotions. Spending time with my dogs helps as well.” (Person 5) - “nothing seems to help.” (Person 6) - “A. Having my Wysa therapist. Before the pandemic people questioned my choice of online therapy but now all therapy is online. B. Staying in contact with family and work colleagues over video chat, phone or text on a regular basis. Setting up shared places online were we can share our experiences. Like a Google Drive, or a Facebook group. C. Doing regular exercise indoors. D. Doing meditation. E. Having a daily to do list and ticking off tasks as I complete them throughout my day. F. Remembering that even though things are bad. It's OK to just have fun sometimes.” (Person 7) - “The coping mechanisms that have worked best for me are keeping a schedule and frequently video calling my best friend, having you here. Having a schedule helps me feel like I’m working towards something and not just stuck at home, and video calling my friend helps me feel less alone.” (Person 8) |
| **Riliv**   - Riliv has initiated #TenangdiRumah (means stay calm at home) with several organizations to promote better well-being during the pandemic. The main feature is to create a special meditation guide to help with anxiety related to Covid-19 - There has been an increase in the number of requests for company subscription to our online meditation since March 2020 - A couple of our users feel more stressed out because they can't leave the house and this causes strain in the parent-child relationship. - One user explains that their stress has increased since being on self-isolation. |
| **Mumsnet**  1) Has there been an increase in user mental health issues that Mumsnet and their moderators have had to deal with since the start of COVID 19?   - Our team has noticed more conversations in which users say they feel extremely anxious or stressed. This arises particularly in conversations about exercise and access to the outdoors, with quite a few users saying they feel that if they weren’t able to access parks or public green spaces their mental health would decline quickly. Conversations in which users discuss the likelihood of a more comprehensive lockdown, such as the one seen in Spain, contain lots of posts expressing this sort of anxiety, even to the extent of people saying it would exacerbate existing mental health problems to the point of suicidal feelings. Anecdotally, our team has observed that these sentiments are most commonly expressed by lone parents with young children. - As you’d expect there’s a lot of anxiety around health (particularly for those who have pre-existing conditions, or loved ones with pre-existing conditions) and infection, with users expressing real alarm about how they can keep themselves and others safe. Users whose children have mental health diagnoses or conditions such as autism are also worried about how they can provide structure and reassurance given the enormous uncertainty and disruption to everyday life. - In response to requests from our users we set up a ‘No Corona Zona’ (a forum in which mentions of the virus are banned) which, among other things, gives users who are anxious or overwhelmed a COVID-free space. We also have a set of protocols for helping users who directly express suicidal ideation or intent, messaging them to direct them to the Samaritans and other sources of emergency support but removing posts of this nature from the forums.   2) Does Mumsnet have any additional statements on the mental health impact on their users of COVID 19 and its consequences?   - One thing our moderation team has noticed since coronavirus became such an all-consuming issue is that users who have long-term experience of coping with mental health challenges have been very generous about sharing their strategies and tips, often taking the lead in comforting and supporting those who have been shaken by the emerging crisis. |
| **MeeTwo**   - “My dad passed away due to the virus 2 days ago and honestly it just doesn't feel real yet” - “[Lockdown has been extremely hard for me as I have a horrible family that doesn’t care about me and treats me like shit My head is stuck with suicidal thoughts and I no longer want to live I have absolutely no one there for me I’m suffering in silence because I have one](https://meetwo.coral.lt/site_admin/application/posts/59333)” - “Thank you all so much, y'all gave me the courage to tell my mom, and she accepts me and loves me. By her response I know my dad will also accept me.” - “camhs has passed me over to another service and because of covid-19 my counselling might have to be video calls at first and that makes me insanely uncomfortable. i hate calling people in my own family but a complete stranger??? no thank uuuuu” - “I love this app but I wish I had someone to talk to in real life, I have no friends and my parents just don’t understand and literally don’t say anything so?? I was ALMOST gonna get a therapist but then corona virus happened and I bet the waiting list is going to be over a year long when everything goes back to normal ugh” - “It is really reassuring when someone else says it bothers them as well because you find out you are no longer alone with this issue” - Within the app we can facilitate conversations between young people who have spectrum disorders, phobias or health issues that ordinarily isolate them. This is particularly helpful for the newly diagnosed: “So I found out 2 days ago that my mum thinks I’m autistic when the lady doing my CAMHS assessment brought up autism (idk why she did but she did) and now I’m on the waiting list to get assessed. I don’t know how to feel about it. It’s hitting me really hard and all I can do is cry at the minute” and after receiving community support: “Thank you @XXXX @XXXX @XXXX and @XXXX You’ve all really helped me come to terms with the situation 💙 - “I am currently living in residential care, my life seems quite unbalanced, I have autism, PTSD, severe depression and due to this pandemic keeping all the kids in the house makes it very noisy and they're very selfish (shouting at me, threatening me because I'm showing symptoms of covid-19) and social services doesn't do anything to help me.” |
| **MIELI Mental Health Finland**  **Mental-chat**  **Mental-chat conversations:**   - clients are worried about how to survive everyday life without social contacts, especially many have talked about their concerns about school / studies and how to manage those concerns about the future (‘will I graduate while schools are closed?’, ‘will I be able to manage all studies on my own?’), loneliness, feeling of being inadequate (‘I am not doing enough’, ‘am I productive enough?’, and worries about old grandparents.   **Mental Gaming:**   - Discussions involve mainly about surviving everyday life, being bored and lonely (‘what will happen to me now?’, ‘how will I manage with school?’ - Some young people are concerned about their mental-health while meetings have been cancelled. After a few weeks it seems like youth on our Discord server has gotten used to Covid-19 as discussions about it are more rare. |
| **Papa Testimonials**   - "I really enjoy my visits with my Pal. She really has been helping me to cope with the current situation we are all facing with this Pandemic. I’ve been feeling stressed out because now my grandbabies are home every day and I’m not used to that. I’m used to them being in school and then coming home after. Now I don’t have any space or time for myself. Today she told me to go into my car or take a walk in my backyard and get some fresh air. She told me I can’t allow myself to become stressed and that I just have to make that time for myself. She even spoke to my grand babies and told them to give me 30 mins to myself because I need that. I always look forward to talking to her. She's just been so great!” - “I was expecting for my regular Pal to call me today but instead a young lady called. We talked and really connected. We talked for about an hour, she was really good. I enjoyed speaking to her very much. I appreciate you guys making this transition. I usually see my regular Pal but I would like to be able to have both of them to assist me with virtual calls.” - “She started talking about my personal self. It was God sent that she called me because we could talk about anything! I’m a pastor's son and usually it’s me checking to see how people are doing. So to see that she reached out to check on me I felt like someone cared. She did amazing! I appreciate you guys for all you’re doing and I look forward to more of these calls!” - “It was great! It was a very pleasant call. She was really enjoyable to talk with. We talked about what I used to before I retired. She talked about what she’s doing right now and talked about her studies. I surely appreciate you guys doing this!” - So surprisingly I was able to talk to my Senior again and he was telling me that he got his medication stolen from his bag so I was able to call his pharmacy and he needed to make a police report in order for them to refill the prescriptions but the police told him that he had to make an appointment but of course I didn’t want him to be without his medications any longer so I called the pharmacy on a three-way and I was able to speak with a pharmacist. However, his health insurance had to override the medication so we called his plan to see if we were able to speak with someone from the Medicare department to override his medication for another 90 day. It was a mission; long story short I was on the phone for about 45 minutes and now he is happy because I gave him some homework which is to call the pharmacy again and tell them that he should be good to go!! - "The Pal asked me if I had groceries and gave me recommendations of places to get free food. She asked me to try to stay home and she even offered to do groceries for me online! We spoke about my family and kids and family in Mexico. We spoke about Mexico and what my favorite things to do there are. She spoke english and I used it to practice but i prefer my calls going forward in spanish. I told her how I learned English from necessity, I took classes growing up and also took classes to get my citizenship in english. She asked me what I do for fun and I told her about my knitting; I make scarves and table top placements. I also told her about how I have been cleaning the garage and between all of this I haven't been bored at all! Thank you Papa I can't wait for my next call in Spanish."   A list of COVID-19 related tasks we ask our pals to perform with our members through our virtual companionship feature:   - Teach members how to use Grub Hub, InstaCart, Amazon, Walmart.com etc. to sustain themselves during isolation without risking exposure at the store. - Teach members how to use their telehealth benefit. - Pick up and deliver medications that have been paid for. - Pick up and deliver food that has been paid for. - Simple welfare checks and light home sterilization. - Address social isolation during this time through virtual services such as texting, phone calls and face to face interaction. |
| **Orygen**  We asked young people what some of the key challenges to working and/or studying online are at the moment, focusing on education and the workplace, but relevant to mental health. Responses with an asterix (*) are likely to be most relevant to healthcare delivery, but many will also impact on mood and MH generally.  **Question 1: What are some of the key challenges to working and/or studying online at the moment?** (from most common to least common)   - Lack of motivation* - Distractions at home - Difficulties with collaborating - Disconnection with team support - Lack of appropriate workspace - Lack of routine - Difficulties with technology* - Increased screen time* - Increased sedentary behaviour - Lack of separation of work-home environment* - Availability of technology* - there is a real digital divide between rich and poor - Availability of work able to be done remotely - Communication problems - Keeping up with deadlines – communication - Lack of immediate in-person feedback* - an issue when assessing and managing risk - Reduced usual functioning*   **Question 2: What are the benefits?** (from most common to least common)   - Reduced commute* - this is a frequent barrier in accessing services so delivery of care online can overcome this - Increased time for other activities - Can go at your own pace - Flexibility of personal schedule* - same as above - Comfortable environment - Improved personal skills - Improved team responsiveness/organisation - Improved technology skills* - Increased attention to student needs - Increased flexibility for those with mobility difficulties – don’t have to go to F2F classes* - will also apply here. - Increased productivity due to less distractions vs. when in office - Increased trust and independence from supervisors - Tight-knit online community – connection in online community   Furthermore these questions, we observed that whilst many young people had embraced telehealth options in the current context, they did note privacy concerns when having these types of consults with family members in the background. |
| **Digital Peer Support**   - A Digital Peer Support Certification was co-produced with scientists and peer support specialists and includes education and simulation training sessions, synchronous and asynchronous technology support services, and on-going feedback. This certification is designed to promote rapid uptake of digital peer support and is designed specifically to address the needs of the current COVID-19 crisis. This certification does not assess a person’s ability to use technology such as zoom, but rather incorporates the latest scientific evidence of digital peer support. This certification includes dyadic training on: Digital Communication Skills; How to Engage Service Users with Technology; Technology Literacy and Usage Skills; Bonding and Connection through Technology; Available Technologies; Privacy and Confidentiality; Monitoring Digital Peer Support; How to Address a Digital Crisis. Since the COVID-19 outbreak, 900 peer support specialists have been trained from 24 different states. (see quantitative section for more details). |
| **National Alliance on Mental Illness (NAMI)**   - Of those who called NAMI Helpline who mentioned COVID-19, the majority of people are distressed and anxious needing support, encouragement and reassurance. Their concerns include finding help, treatment disruptions, accessing resources for family members, financial issues, fear of getting sick, access to medications, tele therapy. - Beginning in April 2020, callers are more often mentioning depression resulting from isolation and despair as the pandemic continues with no specific end date. |
| **Mental Health America**  We asked the question: “What are the main things contributing to your mental health problems right now? Choose up to 3.” Among positive anxiety screeners AND who specified “Coronavirus” ALSO typed in “other” as shown below:   \| - Birth of new baby - Abusive sister - Academics - being away from school - Being stuck with my family - family health issues - Fear of Job Loss - Worrying about my children and my job - Health Issues being delayed by CoVID-19 - I was diagnosed with cancer last year and now I no longer have it. With everything going on in the world I worry about it all the time now. - legal issue - Life - Living situation - course/deadlines - Environment change - PTSD-like episodes from my first panic attack. Lasted over 6 hours and I felt like I was having a really bad heart attack. - Future, Family - Child working on the front lines - Laid off - I don't want to express my feelings because I don't want to worry anyone - my thoughts - Physical Illness - Sexuality - working in healthcare - Mother is in an assisted living facility - I am unable to visit or help with her care as I normally do. - Severe toothache - Thought of loss - University - responsibilities & challenges - stress - Family problems, ambiguous relationship with school - Family - Health issues - Not living with mom \| - Health - Nursing and homeschooling my kids - social situations, social media - life in general - Own health/medical condition - Everything - Being a caregiver - Pretty much everything - work and home schooling the kids - Family - Depression - Life threatening illnesses - Lack of direction and purpose, can't hold a job - Academics - Problem about my study, I am currently making a thesis - changing into online format - Kids and - Divorced and miss my children - Stress - pressure - College and toxic family - family - Stress and family issues - Expectations put on myself by myself or my family, how people view me on a regular basis - home - separation of parents - Social interaction - School - Work events - Work - Potentially losing 2 jobs - Fear of death - Lung surgery - Hearing - Kids - Afraid of losing a parent - being away from family - Chronic illness - every little thing \| \| --- \| --- \| |
| **Mental Health Foundation**  *All anecdotal, all from people living with a psychiatric diagnosis*   - Some people have been extremely distressed when they receive letters describing them as extremely vulnerable, others have been surprised that a preexisting psychiatric condition doesn’t render them clinically vulnerable. - Many have been concerned about breakdown of peer support networks and access to support - People who live with domestic abuse or violence are reporting experiencing high anxiety and stress levels - Some people are starting to voice concerns about the risk of increased surveillance and the potential relaxing of compulsory detention/admission to psychiatric hospital and reduced access to tribunals - Many concerns reported about poor housing, debt and job insecurity - There is a mixed response to social media, some finding it helpful some finding it amplifies distress - There has been some concern expressed that pre planned end of life decision-making is being ignored by clinicians |
| **Sangath in Madhya Pradesh, India: Frontline health workers (particularly in low-resource settings):**   - Community health workers or frontline health workers in low-income and middle-income countries (LMICs) such as India are already overburdened, face high demands and pressures for low compensation, and work in difficult conditions including extreme poverty. - Low socio-economic status, having to travel long distances for work, inadequate incentives, challenging work environments, varying work descriptions and lack of career structure are known contributors to stress, which negatively impact wellbeing and job performance among community health workers. - Given these concerns, community health workers already experience high rates of occupational stress and mental health problems – challenges that often go unrecognized and unaddressed. These concerns have a detrimental impact on their wellbeing and ability to perform regular work duties and quality of care that they provide; - COVID-19 further exacerbates work-related stressors and resulting impact on the mental health and wellbeing of community health workers - In recent weeks, there have been alarming news reports from different regions in India showing the poor working conditions for community health workers, who engage in door-to-door case finding and contact tracing, and play a critical role in containing the spread of coronavirus. For example, community health workers often work without adequate personal protective gear, such as masks or sanitizers, and face fears and insecurities among their patients, as well as added anxieties about the health and wellbeing of their own children and family members. - Community health workers represent the backbone of the health system in India and in most LMICs; therefore, it is an urgent priority to consider the mental health and wellbeing of this workforce as being central to the implementation and effective delivery of care, as well as supporting their essential role on the frontlines in the fight against COVID-19. - Necessary to advocate for frontline health workers, and ensure adequate support in the form of both personal protective equipment, fair compensation, and access to psychological support for managing stress/anxiety are essential - Also consider how digital technologies could be leveraged for supporting health workers – e.g., through support groups on WhatsApp, timely risk communication to avoid exposure to infection, access to real time information and support, etc. - Possibility to leverage technology for offering tailored psychological support services to health workers engaged in contact tracing, and responding on the frontlines |
| **Its OK To Talk**   - Below are themes and anonymised queries submitted by Instagram users who participated in informal “Live” public broadcast sessions hosted by Indian NGO, Sangath’s youth mental health campaign “It’s Ok To Talk” on their Instagram page @itsoktotalk. These sessions lasted 30-45 minutes each and were conducted in English using a “question and answer” format by a project staff member and a clinical psychologist. Sessions were publicised using online posters which were disseminated by the organisation’s social media channels and other online networks. These sessions were initiated as a response to the country-wide COVID lockdown in India that was initiated on 25th March. - The purpose of these sessions, as announced to viewers, was threefold: (i) to help viewers make sense of confusing and distressing thoughts and feelings; (ii) to provide information and tips on managing mental health and wellbeing during the crisis and (iii) to signpost users to local and national resources and sources of support including information, mental health services (web and tele-counselling) helplines or other emergency services such as those to report domestic violence. - Session topics were crowdsourced through open calls via the Instagram ‘Stories’ feature where followers were asked to submit questions and themes of their choice that were having an impact on their mental health. Live session themes were then categorised and summarised by the project team and included: (i) managing worry and ‘uncertainty anxiety’; (ii) strategies to stay motivated while working or studying from home; (iii) practical strategies to manage sleep problems; (iv) dealing with loneliness; (iv) dealing with domestic violence and difficult home relationships; (v) coping with unhealthy habits and (vi) managing love and relationships while physical distancing. - On average, 120 viewers watched each live; and these are currently being held twice per week. A few selected anonymised and paraphrased user submitted questions for are presented below:   **Themes and anonymised queries:**  (1) Managing worry and ‘uncertainty anxiety’:   - This whole situation is just making me anxious — I can’t sleep well at night! What to do? - The numbers of people with Corona is increasing every time I watch the news. This is really making me feel worried. - For those who are currently getting mental health care/therapy/etc. — they can’t meet with their regular mental health provider, what can they do?   (2) Strategies to stay motivated while working or studying from home:   - How can I manage my time when I am at home? - I feel very guilty when I take breaks while at home. How can I reduce this feeling? (worrying about productivity) - How can I reduce my feelings of stress and anxiety about what’s happening around me?   (3) Practical strategies to manage sleep problems:   - I’m finding I wake up in the middle of the night more than usual thinking about this, and find it hard to go back to sleep   (4) Dealing with loneliness   - It is getting overwhelming taking care of my family and working from home, with no break to meet my friends and colleagues. What can I do? - How can I cope with the loneliness?   (5) Dealing with domestic violence and difficult home relationships:   - What should people do during this time when there is domestic violence? - Living with parents, facing verbal abuse, what can I do? - What would be the process to follow for someone who is under 18? |
| **Mentally Aware Nigeria Initiative (MANI)**   - Partnership with Facebook: enables social media users (within Nigeria) to be automatically redirected to receive support services whenever they share any posts that shows they need psychosocial support. - To increase e-social interactions, MANI launched #MyNeighbor Challenge, a challenge where participants will record and share videos of them doing mime choreography of a song. The challenge is championed by a large base of volunteers and open to the general public. - MANI also launched Isolation Journal, an e-calendar filled with daily fun activities individuals can engage in to build resilience, connect socially/virtually with their loved ones, develop on existing interests, and try out new interests. Isolation Journal is also used to document daily activities, keeping in mind to do important things such as eating healthy, exercising, and maintaining basic hygiene; things that usually get very difficult during isolation. The calendar will be in an electronic format and send periodic reminders to users via their mobile phones; inviting them to engage with each activity and share online as appropriate. |
| **Young Leaders for the Lancet Commission on Global Mental Health and Sustainable Development**  Quotes from consultation with the group on how young people are experiencing the pandemic worldwide -- Young Leaders highlight links between mental health outcomes and social, political and economic challenges of the pandemic, and emphasise young people’s resilience and civic engagement:   - “Young people want reassurance - poverty and increased crime resultant from COVID19 lockdown in densely populated cities like Lagos has caused more anxiety and panic than the virus itself” (Chinwendu Ukachukwu, Nigeria) - “There will be more mental health conditions than we can handle. Young people are afraid and hungry at once. There is little or no access to stable electricity and water; most people struggle to survive” (Kumba Philip-Joe, Liberia) - “Young people have been facing profound uncertainty, but have also contributed to information sharing and awareness campaigns, which foster our global solidarity” (Ashley Foster-Estwick, Barbados) - Young people in KwaZulu-Natal feel great uncertainty and anxiety of the future — they are increasingly depressed and feel incredibly de-motivated in their studies and find it difficult to work in isolation — away from classmates and friends (Chantelle Booysen, South Africa) - “Young people’s anxiety and fear during this time has its roots in violence and police brutality, as well as concerns around our already strained health system. Our campaign’s online webinars are initiating a movement for compassion, hope and social connection during this challenging time” (Damian Juma, Kenya)‘ - “We are seeing how young people are wanting to be supported right now and seeking to gain from their friends - but often they lack the tools and skills to provide effective support” (Lian Zeitz, USA) - “Young people are confused, afraid and uncertain. Even though the country has few declared cases of COVID-19, it is now becoming clear that the government has been covering up COVID-19 cases and failing to communicate the severity of the virus” (David Karorero, Burundi). - “The government has been incredibly proactive, but the mental health effects are severe, especially as the crisis coincides with our national mourning period. Young people are driving mutual aid initiatives to support the more vulnerable members of society” (Grace Gatera, Rwanda) |
| **Consultant NHS nurse:** Insights collected by Emma Selby from anonymous user and staff feedback/messages all whom have consented to share and have their feedback and edited down if needed:   - “Since the lockdown started I've been finding things extra difficult. Lots of people are making jokes that you will either leave lockdown a domestic goddess or obese but i dont think they relaise that for people like me who suffered from Anxiety before lockdown the closures of services and support has been really difficult. I was finding everyday really difficult and getting strong urges to self harm. I ended up using a combination of [digital tools] to help me resist urges but also to explore how I was feeling and come up with a plan. I have more structure to my day now and that’s really helping” - “Two weeks after the government announced lockdown I went into labour four weeks early with my second child. I had a really strict birthing plan as i had a traumatic first birth and i was terrified but it all went out of the window because of limits on visiting etc. I think i rushed to make myself appear well so we could get home to my partner and some support but i had massively underestimated how hard it would be without being able to have my mum over for support and not being able to share these initial days. I began feeling really low and like i had been robbed of something. My midwife recommended [a digital app] and i’ve started using it as a place to talk about the things you cant really talk about with other people who just insists on reminding you how lucky you are. I am feeling better now and we skype my mum everyday to help baby get to know her.“ - “My Husband suffered really badly with health Anxiety and OCD and was seeing a private counsellor last year and had gotten to a really good place when the outbreak started. His hands started to bleed he was washing them so much, he would force the children to keep their hands in their pockets if we went out and he wasn't sleeping. We couldn’t access support anywhere as no where was seeing anyone so we tried [a digital sleep app] just as something to try. We learnt some really helpful tips that our whole implemented which is helping us all!” - “I see a lot of people posting messages about all the wonderful things they are achieving in lockdown or the benefits to the earth and that is properly wonderful but i think we also need to add a dash of honesty. Lockdown is hard. Not being with loved one’s is hard. Being with loved one’s 24/7 is hard. Lack of routine is hard. Finance worries are hard. Getting out of bed is hard. Going to bed at a sensible hour is hard. Pandemic’s as it turns out are Hard and that’s okay. I have found that not being able to work out as much, go swimming or climbing, has been really hard for me as it’s how i deal with my mental health and i felt really bad about asking to increase my antidepressant dose a bit during these times but then i was reminded that it’s okay to find hard times hard and social media doesnt know what's best for me i do.” |
| **Money and Mental Health Policy Institute**   - Many of the 568 respondents to our survey noted the toll the spread of coronavirus had already taken on their mental health. For some, this was through increased anxiety about their health or that of their friends and families. “My anxiety has been through the roof, I've been having panic attacks. I'm still going out to the shops but have developed a fear of contamination that makes it very anxiety inducing. I was having counselling but that has been stopped, which is a blow, especially at a time when my mental health is suffering so badly.” - The vast majority of respondents agreed or strongly agreed that as a result of coronavirus they were worried about struggling to access mental health services if they needed them. Some respondents also described finding it harder to access support from friends and family with social distancing measures in place. - “I have been told that I will lose my job. I have been on reasonable adjustments within my organisation, while recovering from depression. The role I am doing is to be discontinued because the company has decided it can no longer ‘carry’ people. I am devastated at the actions of my employer of 15 years.” - Many respondents had already experienced an income shock, such as job loss or, or were concerned that their financial situation would deteriorate and this would affect their mental health. Some respondents raised concerns about the level of support offered through the benefits system and the accessibility of this support. - *“If I self-isolate I will only be paid SSP [Statutory Sick Pay] and this isn’t enough to cover my monthly outgoings.”* - *“I'm terrified, it's scaring me and my anxiety and paranoia has gone through the roof. I'm worried because I have PIP [Personal Independence Payment] forms to send back and CAB [Citizens Advice Bureau] is closed for appointments due to COVID so I can't get help.”* |
| **Turn2us**  We are currently surveying all our users with the following questions:   1. *How do you think that Coronavirus will affect yourself and your family?* 2. *What are you most concerned about at the present time?* 3. *What would help you and your family the most at the present time?*   In terms of impact on mental health, the main worry for the majority of respondents was being unable to afford rent, food and other essential bills, such as heating. As with the previous question, it seems even for people who have been offered a furlough (80% of their wages), they still have financial worries. Financially, users are worried about the risk of losing their home or becoming homeless and they are also worried about the longer-term financial impact on their family:  *“Depending on how long we are unable to work, we could lose our home. we are struggling financially, serving on loans from family buy food and pay bills. Even when this crisis is over,it will have a lasting financial impact on us and will take us a long time to get back on our feet”.*  **Being alone:**   - But worrying about how to afford necessities was also linked to a key concern about being alone during lockdown and social distancing. The data suggests people are struggling to be alone in different ways; from having no support network at all (2), or being the sole responsible adult for other people (5). Another particularly isolated group are people who may not have their family living in the country (4). Whilst there are concerns over how being alone is going to affect them financially (1)(4), there are also concerns around the loss of social interactions (2)(3)(5). - *“I am a single parent currently on zero income and have fallen through the net on all government help" (1)* - *I live alone and dont see anyone” (2)* - *[I am] shut off from things that help me through my week” (3)* - *"I ill need some help to pay rent and food as i am single person and dont have family over here” (4)* - *“WORRY OF BEING A SINGLE PARENT AND CHILDS FATHER IS IN HIGH RISK CATAGORY AS ARE GRANDPARENTS” (5)*   **Single parents:**   - Single parents are seen as users who are particularly vulnerable during covid, both because of the impact on their income and also their support network. This is particularly the case if they, or their children, are in an 'at-risk' group for the virus: - “*I am a single mother of two young children. No family to help. Youngest is three with severe asthma who has been hospitalised annually with respiratory problems ie pneumonia. So I am struggling to get food as do not want to risk my children’s lives taking them into a store but now unable to get delivered groceries. Very worried (1)”* - “*It has affected us as I got very ill and am a single parent, meaning that I was unable to properly care for the children while so Ill. Also I have developed panic attacks and generalised anxiety which is impacting on my ability to work efficiently (2)”* - “*I’m in the very vulnerable group. I won’t be allowed to leave home which means my daughter will need a carer and extra support if she wants to go out (3)”* |
| **IncomeMax**   - Since COVID-10 there has been an influx (step change) of new customer groups that have never had to rely on welfare or government support (e.g., have previously been self-sufficient). - Some ex-employers are being supportive when, for example, someone handed in their notice just before COVID-19 lockdown (i.e., as long as they remained on the payroll they could be furloughed to go back to the employer).   **DWP Twitter posts** (non systematic selection by domain experts, IncomeMax)   - What about the thousands who started new jobs to better ourselves after the feb 28th cut off and before the [#coronavirus](https://twitter.com/hashtag/coronavirus?src=hashtag_click) hit the UK but now sit **suicidal** in the gap entitled to nothing despite being lifelong tax payers? [#newstarterjustice](https://twitter.com/hashtag/newstarterjustice?src=hashtag_click) [#newstarterprotest](https://twitter.com/hashtag/newstarterprotest?src=hashtag_click) [#newstarterfurlough](https://twitter.com/hashtag/newstarterfurlough?src=hashtag_click) - **we are .........dead.....no money no food**......4 weeks in isolation UC no answers....i have no other way to provide for my children and i don`t care about the bills....i will have to go out and improvise something. - Yeah exactly I agree I had my PIP stopped wrongly with the appeals now have been delayed which is very convenient I now living on not a lot and **done know how I'm going to.feed my self** or pay for my utilities due to my **complex health and disability,I will be taking up a NHS bed!** - What about the thousands who don’t qualify (I’m a student) and have changed jobs after the 28th of February? I’ve paid tax, NI and contributed for many years before I decided to study and I’m left with a small student loan to survive now. **You have sentenced me to poverty** - Got my letter yesterday to tell me it’s being taken away. The welfare system has kicked me when I’m down already, **made me physically ill** & caused a flare up of my health just when I don’t need to go to a hospital mid pandemic. - You’ve taken my mobility allowance away at a time I can’t call upon others for help, even a hug at the **immense stress** i’m now under as a single@parent with **multiple health issues**. I’m not sure how my EDS & POTS have gone away magically since I was awarded it last year. - have been trying to get through for 7 days, no luck over 6 hours spent on the phone waiting in total, and today once again it says call back another time, **what are we supposed to do**? - Over 7 days trying to get through. Over an hour every day. All to close a claim. Still can't get through and **anxious** about my situation now. Absolutely abhorrent service. |
| **Anonymous Financial Services Provider:**  One large lender/financial services organisation reported:   - “A recent increase in suspect transfers into accounts from a normally trusted source has flared tempers. Banks can freeze accounts that receive suspect transactions to prompt contact with customers to support an investigation, but cannot tell customers when an account is frozen for this reason, as this could constitute “tipping off”, a criminal offence itself. Customers impacted, on attending branches and not getting the answers they seek, have reacted in varying ways – and in this environment of heightened stress on all sides, staff have had cause to call police to branches to help address threatening customer behaviour. Whether this is stressed out staff who have a heightened sense of risk, or stressed out customers worried about access to their money and with less access to support (longer wait times at contact centres, shorter branch opening hours) isn’t clear – but the activities of fraudsters are able to have a much greater impact into today’s unusual environment.” - “A well meaning advert or internal comms piece, intended to show solidarity and support, has triggered a response in branch colleagues who are having to work in PPE, interpreting the vague government guidance on social distancing to the best of their ability – the majority of their colleagues being able to work from home (which, while it has its own challenges, is perceived by those on the front line as easier and less challenging).” |
| **Healthy Virtuoso**   - Most users are trying to find any way to do sports and stay active. We believe they really miss endorphins as we have been seeing examples of people running up to 200 times around their house blocks. - There has been an incredible increase in the usage of home training (video tutorial), yoga and meditations apps that we have tracked from a lot of users. |
| **The Mind and Soul Foundation**  “...The resultant uncertainty [COVID-19] has acted as a catalyst to renewed religious practice, particularly prayer. Search engines like Google have recorded exponential increase in page requests for prayer, approximately doubling with each 80,000 new global cases. Uncertainly has historically been relieved by religious practices, and after a period of extended stability we are seeing a return to dependence beyond personal confidence in medical provision and into the divine.” Rev Will Van Der Hart |
| **Dark Web (insights by The TellFinder Alliance)**   - The child sexual exploitation offender community on the dark web has been taking advantage of the increased number of kids online and the increased amount of time they are spending on various online platforms. Offenders are adapting their grooming strategies to target newly trending platforms (e.g. Zoom) as well as discussing the increase in "soft" material, i.e. parents posting more pictures and videos of their young children as they spend more time at home with them. There is also an observable change in pattern-of-life as many offenders are able to spend significantly more time on their computers during quarantine. The evidence here is primarily anecdotal, as there is not yet enough data for robust quantitative analysis. - Law enforcement investigators are on heightened alert and are continuing to go into houses for warrants, arrests and child rescues, at the risk of COVID exposure. |
