# Supplementary material for: Early Warning Signs of a Mental Health Tsunami: A Coordinated Response to Gather Initial Data Insights From Multiple Digital Services Providers
Source: Front Digit Health. 2021 Feb 10;2:578902. doi: 10.3389/fdgth.2020.578902 (PMC8521957; doi:10.3389/fdgth.2020.578902)
Supplement: Supplementary file 3 [file Table_3.docx]

**Table 3: Quantitative data insights**

| **Quantitative Insights** |
| --- |
| **Ieso**  Referral numbers:  In the weeks since the lockdown was announced in the UK, Ieso Digital Health saw an 84% increase in referrals to our service, relative to the same time period in 2019. The patterns of change in referral numbers, and particularly referral pathways (i.e. service vs self-referrals), are complex and dynamic, and difficult to interpret at this early stage. We will continue to monitor and explore these data in more detail during the next stages of this crisis, including research on the impact of the Covid-19 on other variables, such as clinical presentation (diagnosis, symptom profile and severity), average treatment duration, and clinical outcomes.  Patients’ clinical presentation:  Ieso Digital Health routinely uses natural language processing and machine learning models to quality control care delivery and to assist with service delivery operations, such as therapist allocation to patients. In the context of this work, we have observed changes in patients’ language as they present to the service. Patients describe the mental health difficulties they are experiencing and the reasons bringing them to therapy in a self-assessment questionnaire. We have observed that 20% to 30% of patients are mentioning Covid-19 in this questionnaire. While this does not necessarily indicate that 20% to 30% of patients are having mental health difficulties caused by the Covid-19 crisis, it clearly suggests a significant impact of Covid-19 on patients presenting with mental illness.  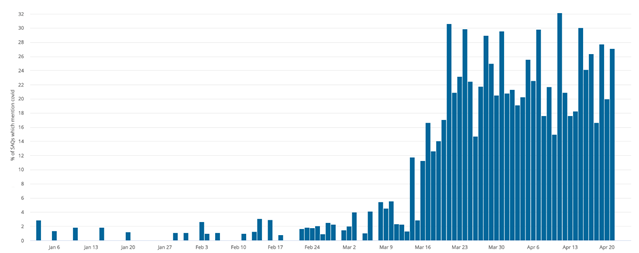  **Figure 1** – Increase in percentage of self-assessment questionnaires mentioning Covid-19 related words with time, for the first quarter of 2020.  Worry themes in therapy:  Ieso Digital Health has also explored the impact of the Covid-19 crisis on patient language within therapy sessions, and specifically on worry themes mentioned by patients. During March 2020 we observed a significant increase in worries about viruses, including coronavirus and Covid-19. Approximately 15% of worries in therapy sessions in March were about viruses, coronavirus or Covid-19. This compares to approximately 40% of worries about family, and 5% of worries about finance, which remain stable over time.  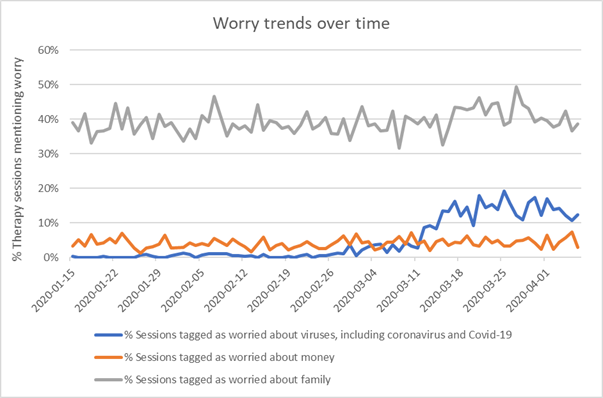  **Figure 2** – Percentage of different worry themes in therapy sessions mentioning worries, across time, for the first quarter of 2020. |
| **Babylon (UK clinical operations only):**   - We have had more concerns about child safeguarding flagged by our clinicians in the past month; there has been a 35% increase in concerns reported. - In March 2020 21.1% of all appointments conducted in the UK were for COVID related problems with an almost even split between NHS and private cases. - Significant increase in demand for both private and NHS services digitally but supply matching demand at present - 4.2% of all coronavirus cases are coded as "patient concerned about covid-19" however not clear if related directly to mental health - Since 20th January 2020 we have had over 20,000 coronavirus related appointments; this can vary from questions about self isolation and social distancing to concerns about symptoms |
| **Vala Health**   - 3 times increase in our registered profiles base from 10th March to 8th April - General booking volume was approximately 5 times the normal volume, with a doubled volume of mental health related consultations. - We saw general health queries tail off in about week 4 of the lock-down to almost pre-covid levels, but our mental health volume continues to rise. - Our GPs reported 4/10 appointments were ‘Covid’ related during this period, but just 20% of those Covid related appointments actually mapped to a potential Covid diagnosis (referral for a test) and 80% were likely symptoms of stress and anxiety (short of breath, elevated heart-rate, increase in potential temperature, but not fever) - Our mental health related case work also increased significantly over that period, with an increase in requests for mental health related medication, however, we believe that was people ‘panic stocking’, and in most cases our GPs talked the member into an alternative strategy - Statistically all four of the patterns above slowed down to what we would classify as a ‘normal’ level at the beginning of April. |
| **Ooca**  The number of video counseling sessions increases 229% compared between Feb-Mar 2019 to 2020 and the usage surge 70% in Feb20 compared to Jan20. About 10% of sessions is rateled to COVID19 topics directly. |
| **Kooth (Kooth & Qwell)**   - Evaluated 2 sets of data across both Kooth and Qwell platforms. Comparisons between 3rd March - 4th May 2019 and 1st March - 2nd May 2020 - Demand has increased by +37% during the period of the pandemic, compared to the same period last year, demonstrated by logins to the service.   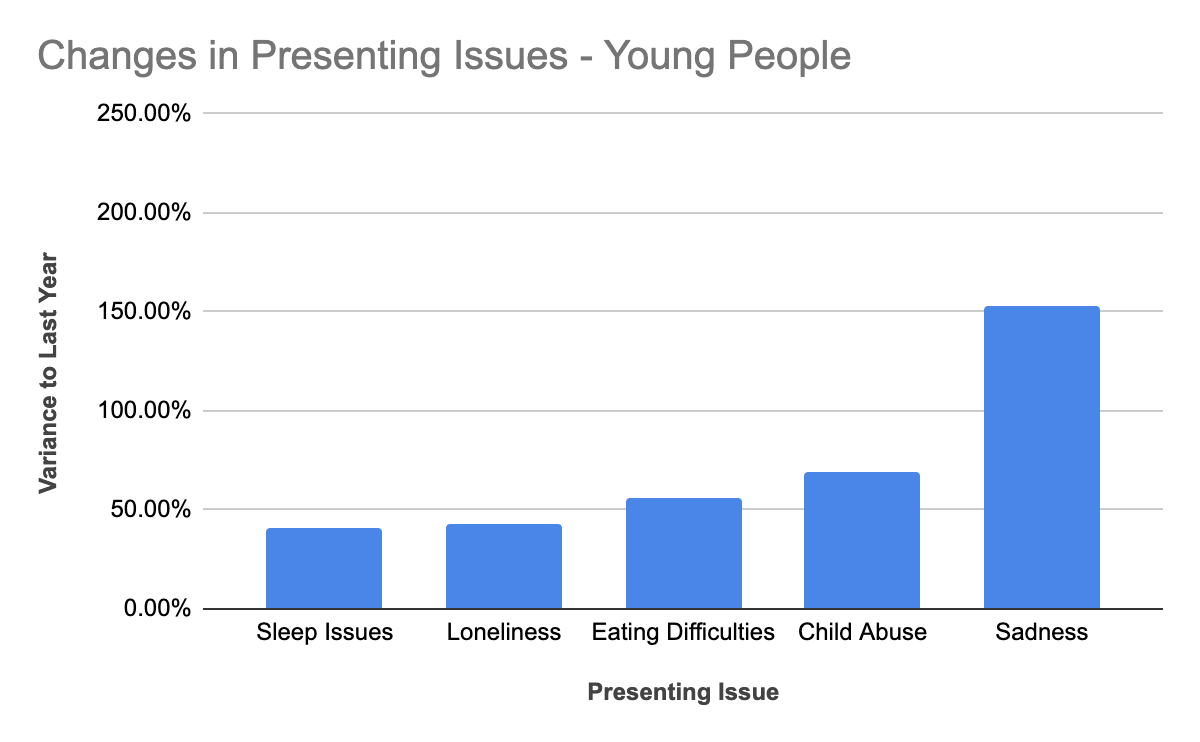   - Young people (aged 10-17) have shown a significant increase in particular issues they are presenting during counselling and other interactions with the service. Issues relating to child abuse has increased by 69%. Sadness has increased 153% compared to the same period last year, eating issues are 56% more, sleep issues +41% and loneliness +43%. - More information on the issues being presented by young people, with regional trends can be found here - <https://xenzone.com/wp-content/uploads/2020/05/150520_CYP_infographic_D4.pdf>   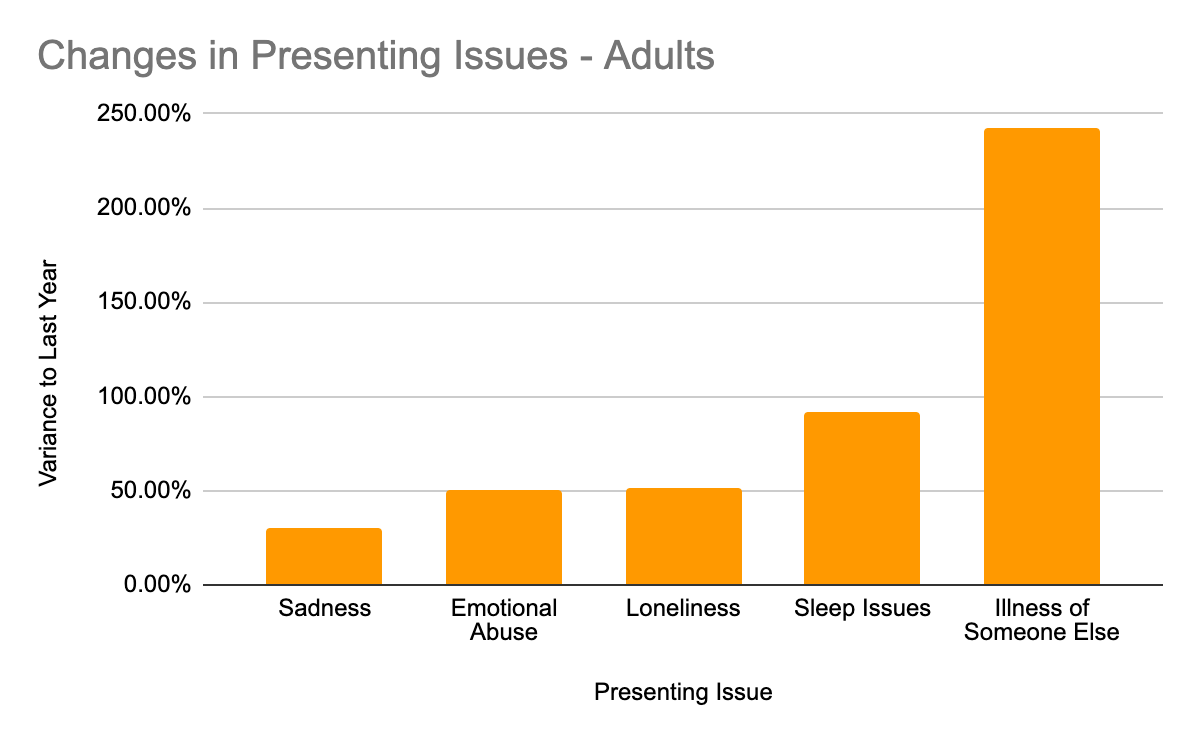   - Adults aged 18 years and over have shown significant increase in the following issues being presented. Issues relating to the Illness of someone else have risen by 242%, sadness has increased by +30%, loneliness by +51%, issues relating to sleep +92%. Issues relating to emotional abuse have increased by 50% compared to the same period last year. - More information on issues relating to adults can be found here - <https://xenzone.com/wp-content/uploads/2020/05/150520_Adult_infographicD4.pdf> - Presenting issues are logged against young people and adults when they present issues during chats, messaging and any other interaction with the service. The proportion of people presenting specific issues have been compared against the previous year. This means the comparisons are not skewed by increased demand, and show true shifts in issue frequencies. |
| **Silver Cloud Health**  **Changes in numbers of clients using online mental health versus before COVID-19**  The following table displays the change in users in the largest 25 NHS providers across England and Wales that use SilverCloud for iCBT services. The data is accurate as of 27/04/20, (i.e., the final numbers for April may increase):  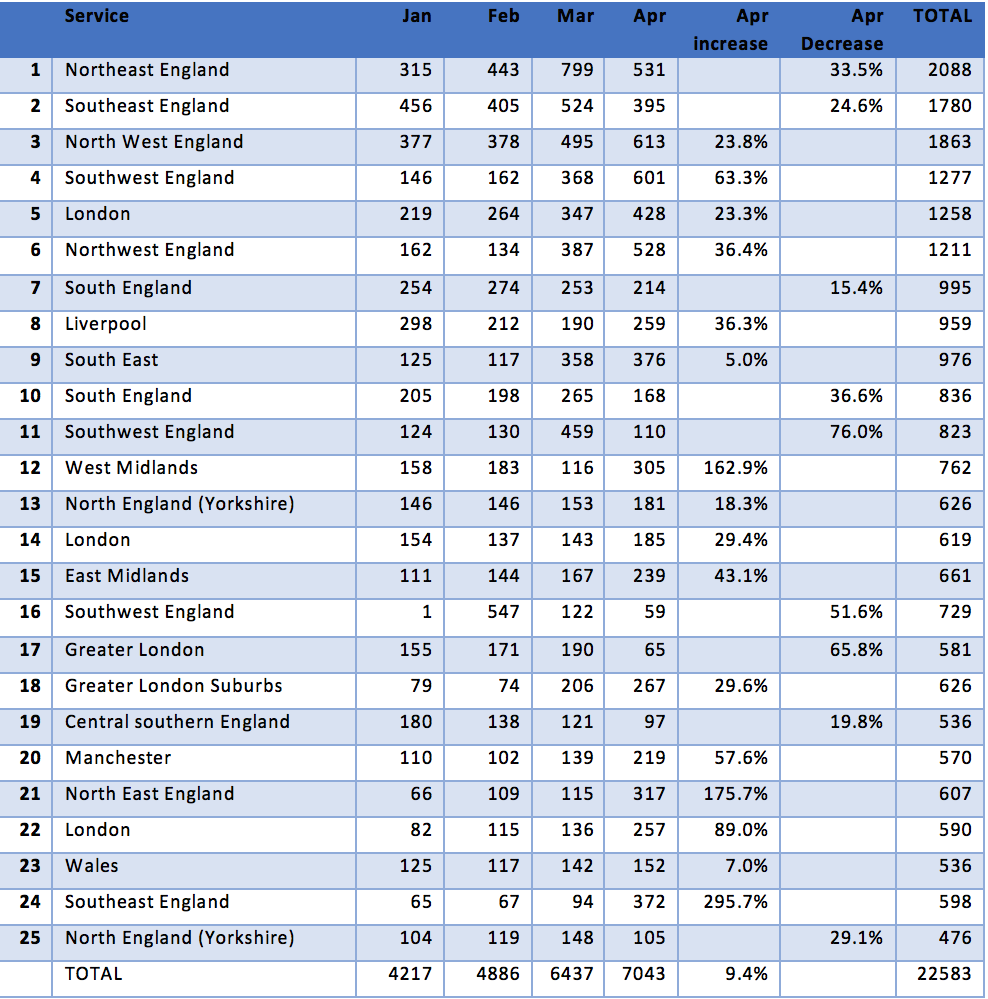  **Changes in symptoms at mental health services (UK IAPT)**  Exploratory analysis of our data shows that there is as yet no evident increase in the level of symptoms of depression and anxiety across services, or indeed when focusing the analysis on some of the larger services. This is true both when comparing mean symptoms scores for March and April of years past (2017-2019) compared to 2020, and when observing trends from the past 12 months. It is difficult to make any broad conclusions from this, only that the profile of users assigned to iCBT within UK IAPT services remains largely the same. Yet there is the likelihood that these will change over time as the impact of COVID-19 takes hold.  **Changes in usage at mental health services (UK IAPT)**   - Data from 3k+ users in the month of April 2020 show that the average time spent on the platform for each login has increased with respect to the same time last year, with users spending an average of 4min more each time they login. This is especially evident in programs such as the Space from Anxiety program. In April 2019, users were spending an average of 16.0 min each time they logged in to the platform [95% CI 15.0-17.1). Now in April 2020, the average time spent per login has significantly increased to 21.5min [95% CI 18.6-24.4].   **Further Observations:**   - Across IAPT, the adjustments to lock-down took some time and there is evidence that service levels for digital treatment are in fact increasing. SilverCloud Health who serves 70% of IAPT services noted a 10% increase in use of digital treatments for the population (see table above), right across the country. - Whilst overall the number of users in the last month has increased by 10% (likely to be closer to 20% when final figures for the month of April come in), some services have already seen a two or even three-fold increase in users from March to April. - The observed 10% increase in rates of new users amo - ng NHS providers in the UK may be a preliminary indication of how services are evolving and responding to the first sights of the pandemic in terms of mental health care. - Users are engaging more with the programs, which might be an early indicator of an increased focus on solutions for mental health difficulties and stressful factors, as they relate to the current pandemic. - The rapid development of new content emphasizes one key advantage of digital interventions, which is the capability of rapidly deploying new content. This is especially relevant in a rapidly evolving situation like this pandemic, where the mental health needs of the population may evolve and change over time. |
| **CBTClinics**  Pre-COVID-19   - 60% of patients chose in-person f2f therapy sessions - 40% of patients chose digital video, audio or online therapy sessions   Peri- COVID since lockdown   - 100% digital video, audio or online delivery now being provided. - 13% of all F2F patients have been unable or unwilling to move to digital delivery due to their personal circumstances (technology, living conditions, unwillingness to engage remotely). |
| **Minddistrict**   - The number of minutes used for video calling sessions between professionals and their clients using the Minddistrict platform has multiplied by 80 times when compared to before the Coronavirus crisis - There are now 3 times as many professionals and clients using the Minddistrict platform compared to before the Coronavirus crisis |
| **Big Health**  Data is majority NHS staff which has had 4 weeks to collect data from launch. Social care has had less than a week from launch. Baseline data collected from health and social care staff indicate that this population has mental health need:   - - **Sleepio data, n= 6,904**   - SCI-2 (sleep condition indicator) was 2.92 out of 10.   - PHQ-2 was 2.06 out of 6.   - GAD-2 was 2.63 out of 6.   - **Daylight data, n= 2,196**   - SCI-2 was 4.3 out of 10.   - PHQ-2 was 2.82 out of 6.   - GAD-2 was 4.21 out of 6. - Note SCI-2, PHQ-2 and GAD-2 are established clinical screening tools. |
| **Qare**  **Teleconsultations with a psychiatrist or psychologist:**   - Number of teleconsultations with a psychiatrist increased by 382% in March 2020 compared to February 2020 - Number of teleconsultations with a psychologist increased by 195% in March 2020 compared to February 2020 - The main reason was anxiety   **Mon Sherpa (personal chatbot assistant for mental health issues):**   - Downloads increased by 201% in March 2020 compared to February - Daily Active Users increased by 133% in March 2020 compared to February - Average number of sessions per active device increased by 7% in March 2020 compared to February - A majority of users seeked help linked to the current lockdown in France, of which 30% regarded sleep issues   COVID-19 accelerated our growth, which was already quite substantial before the pandemic. |
| **Biobeats**  This analysis is conducted on 102 users showing enough data to be included into the analysis, i.e. the users that have more than 60% of Heart Rate (HR) data recorded (more than 86 Heart Rate observations to a maximum of 144 per day - the HR data are recorded every 10 minutes) during the investigation period. The time period analyzed is from March, 7th to April, 14th.   - **I**n our cohort, sleep quality deteriorated versus pre-COVID19: the number of awakenings during the night continuously increased from the first day of March (number of awakenings = 0.5738 + 0.0049 * day_number; r2 = 0.11; r = 0.34). Moreover, the total time awake and consequently the mean of the time awake during each awakenings phase are longer than the days before the lockdown. The longer time awake detected during the night suggests that users sleep worse due to the stress stimulus induced by the isolation. Heart Rate Variability (SDNN24) was affected in the first week of isolation (low variability), probably due to the high stress perceived from users induced by the change in their habits. |
| **Unmind**  **Analysis 1:**   - For this particular group analysis, our sample size was n = 274. We conducted within-subjects group comparisons using the Unmind Index (a bespoke self-report measure of mental health and wellbeing validated in the UK against the GAD-7, PHQ-9, CORE-OM and WEMWBS) between the periods 7th October - 31st December 2019 (pre-Covid) and 16th March - 21st April 2020 (peri-Covid). - Between pre-Covid and peri-Covid timepoints, anxiety levels significantly increased (*p_FDR_*<.001). Interestingly, users also reported significantly higher subjective evaluation of, and satisfaction with, their physical health in the peri-Covid period (*p_FDR_*<.01).   **Analysis 2:**   - In a separate analysis, we compared the Index scores of healthcare workers (*n*=11,849), to those of a non-healthcare user group (*n*=9,854) for the period 16th March - 21st April 2020. - Compared to other sectors, healthcare workers scored lower across the Index overall. Compared to the rest of our user base, healthcare staff report feeling less able to cope (-13.9%; *p_FDR_*<.001) and less able to manage anxiety (-21.6%; *p_FDR_*<.001).   **Additional:**   - Within our day-to-day mood tracker, the frequency of users identifying anxiety as one of their primary emotional states was 38% higher in the peri-Covid time period compared to pre-Covid. |
| **Expert Self Care (distrACT app)**   - iPhone downloads in the last 90 days: We noticed a 359% increase in downloads (5.36K) compared to downloads in the preceding 90 days (1.17K). Most of these were on or around 11 April 2020. Territory: 5.18K of 5.36K downloaded within Europe. Of these, 5.15K were in the UK. - Android downloads in the last 90 days: 762 (24% increase to the previous three months). |
| **Alpha Health**   - Negligible increase in stress from March to April. This was statistically significant in Spain, but not in the USA or UK. - More than half of our active users (56.8%) engaged with the Covid-19 content - Due to a parallel marketing campaign, we are unable to make any conclusions regarding increased demand during the Covid-19 outbreak. |
| **Neurum Health**  Mood journals are a form of ecological momentary assessment, which captures users’ mood and contextual data to understand certain risk factors and triggers in their natural environment.   - 9% of all mood journals submitted were directly flagged as COVID-19 related between Feb-April 2020. - 80% significant average increase in negative sentiments recorded on the app between January-April 2020. - 8.1% significant average increase in users reporting stress detected between Feb-April 2020 - 67% significant increase in users reporting anxiety in their mood journals between March-April 2020. - Top contextual factors tagged to negative sentiments in mood journals:   - February 2020: Relationships (39.29%), Work (21.43%), and Sleep (14.29%)   - March 2020: Work (27.27%), Self (22.01%), Relationship (13.40%)   - April 2020: Work (25.56%), Self (22.04%), Relationships (15.02%)   Our team did our best to parse out inputs that may have been related to the protests at their height in Hong Kong between June 2019 to January 2020. |
| **NHS London** [**IAPT**](https://www.england.nhs.uk/mental-health/adults/iapt/) **Clinical Leads**   - In the initial weeks of March 2020 as the lockdown was announced in the UK, IAPT London Clinical Leads reported local service decreases in referral numbers of up to 50% in some boroughs (see qualitative section for more details). |
| **ORCHA Health**   - Compared to the same time last year, ORCHA has experienced a 181% increase in page views (52,801 to 148,604), a 279% increase in users of the site (8,104 to 30,674), and a 258% increase in condition specific searches (8,275 to 29,665). - Specific to mental health, ORCHA have observed an 86% increase in searches for apps for the treatment of anxiety, a 176% increase for apps dedicated to the management of depression, a 328% increase in searches for apps related to sleep, and a 7500% increase in searches for health-apps related to the prevention of self-harm. |
| **Public Mental Health Services South Australia, Clinical Leads**   - Number of clients referred to [IAPT service](https://www.sahealth.sa.gov.au/wps/wcm/connect/public+content/sa+health+internet/health+services/hospitals+and+health+services+metropolitan+adelaide/flinders+medical+centre/services+and+clinics+at+flinders+medical+centre/services+at+flinders+medical+centre/improving+access+to+psychological+therapies+%28iapt%29+services+at+flinders+medical+centre) have increased by 30% from 17th March - 20th April when compared to the same period in 2019. Notably, the proportion of referrals coming from the Emergency Department has nearly doubled from 25% to 58%. Therapists subjective feedback suggests an increase in chest pain/psychosomatic cases. - Early insights from [sixteen regional mental health services of South Australia](https://www.sahealth.sa.gov.au/wps/wcm/connect/public+content/sa+health+internet/services/mental+health+and+drug+and+alcohol+services/mental+health+services/mental+health+services) suggest people with severe mental illnesses are having less frequent contacts with mental health professionals as a result of Covid-19. Since 12th March 20 when COVID-19 restrictions were introduced the number of contacts made by case managers with patients has almost halved when compared to a similar period last year (12th March 2020 to 24th April 2020 vs 12th March 2019 to 24th April 2019). During this period the proportion of contacts made remotely (telephone, videoconference) has increased by 20%. |
| **Wysa**   - Wysa witnessed a 77% increase in new users during February-to-March 2020, as compared to the same period in 2019. - The proportion of users who referred to COVID-19 during therapist sessions increased week-on-week during March 2020, from 5% in the first week to 60% in the fourth week. - Usage of digital self-help tools related to stress/anxiety, sleep, relationships and Covid went up by 55%; from 49% of total tool engagements to 76% of total, between the 1st and third week of March 2020. |
| **Owlie**   - +10% Total Users (organic acquisition) in the period March 16th - April 22th 2020 (beginning of the containment period in France). - +100-500% Daily New Users since the announcement of the containment in France compared to February 2020 - +50% Daily Active Users in March compared to February - Compared to the same time period in april 2019, there was a 50% increase in the number of Owlie's users. - The most frequently used modules during this period are crisis management and therapeutic tools for anxiety |
| **Riliv**  There has been an 80%-85% increase in demand for both online counseling and subscriptions to online meditation February - April 2020 (compared to January 2020) |
| **StopBlues**  During the lockdown period (from March 17 to April 22 2020), StopBlues has witness:   - 146% increase in the total number of users per day during the period as compared to the same period in 2019 (24,787 compared to 10,049). - 162% increase in users who created an account for using StopBlues as a supporting aid compared to the same period in 2019 (24% in 2019 to 64% in 2020). - Longitudinally, when comparing trends over similar intervals of time (36 days) before and after the lockdown start date (17/03) in 2020 and 2019, the increase in users who created an account for getting support is 5 times higher during the lockdown period (+65%) than it was over the same periods in 2019 (+13%), showing a greater need for support during the pandemic period.   Overall, StopBlues users rate their average mental health as fair to poor:   - There are no significant changes between the lockdown period and the same period in 2019. However, the overall Mood followers’ scores at the start of the pandemic in France in March 2020, were slightly worse than in March 2019 (-6% for spirit). They also followed an opposite trend: while they improved from Feb to March 2019, they worsened in 2020. - The drop in scores among new users creating an account at the start of the pandemic in France in March 2020 were 3-4 times greater with a 20% decrease in moral/spirit, 4-5 higher in energy with a 9% decrease and 2 times higher in overall feelings with a 11% decrease - In April 2020, all users were better on all these indicators. (We do find the same trends between March and April 2020 in a qualitative cohort of 60 people that we interview every two weeks since the beginning of the lockdown period)   General Anxiety Disorder-7 (GAD-7) and Patient Health Questionnaire-9 (PHQ-9):   - Two-thirds of users show moderate to severe anxiety (Score >10 ) in GAD7 and this proportion is relatively stable in 2020 (over the same period in 2019) - Around 50% of users show moderately severe and severe depression ( score >15) and this proportion is relatively stable in 2020 (over the same period in 2019). Sample size: GAD 7 users (762 first quarter of 2019; 566 first quarter of 2020); PHQ-9 users (794 first quarter of 2019; 562 first quarter of 2020) |
| **BeyondNow**   - 53% increase in downloads of its suicide safety planning app Beyond Now from February to March 2020, compared to a 17% increase from January to February 2020. - Monthly downloads for Beyond Now differ across seasons and are usually highest during the winter months in Australia, from June to August. Downloads in autumn months (March to May) are, on average, two-thirds of the downloads recorded in winter months. Downloads for March 2020 exceeded the winter average by 9%. |
| **Spill**   - Website traffic more than 2x any previous month - More business enquiries for the Spill product in the last 2 months (Mar-Apr) than in the previous 2 years - +188% active users of the Spill Slack app in mid Mar - mid April vs. previous 30 days - +171% increase in questions asked to therapists over message in mid Mar - mid April vs. previous 30 days - +446% increase in number of therapy sessions in April vs March |
| **Mumsnet**   1. **Survey results:**   *Date: 27th - 30th March; Number of respondents: 1,398*  61% are concerned about their own mental health during lockdown  67% are concerned about their children’s mental health during lockdown  49% are concerned about their partner/husband’s mental health during lockdown  *Date: 3rd - 6th April; Number of respondents: 1,016*  65% are concerned about their own mental health during lockdown  69% are concerned about their children’s mental health during lockdown  53% are concerned about their partner/husband’s mental health during lockdown  69% feel more stressed that they usually do  61% report that their sleep is more disturbed than it usually is  72% are concerned about their family’s mental health during the coronavirus outbreak and the lockdown   1. **Mumsnet graphs showing the changes in use of key mental health related terms:**   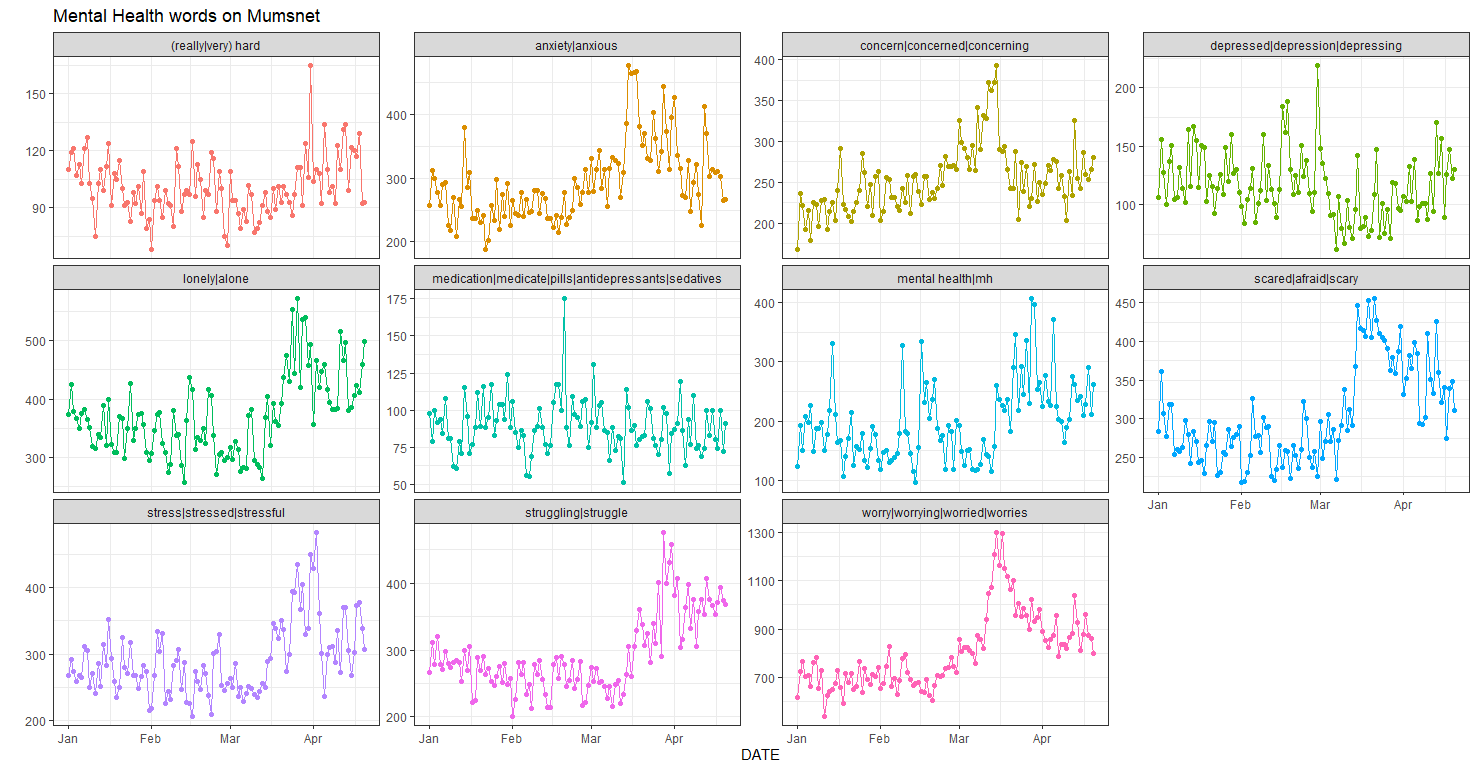  **Key findings from plots above:**  1) Moderate mental health words are more likely to have seen an increase (eg struggling, worry, scared, alone, mental health) than stronger terms (anxiety, depression, medication). This may be for a number of reasons - users might be more likely to understate or not discuss their specific conditions  2) There are two different peaks: 1) the middle of March when social distancing started - here we see more expectant words worry and scared and 2) the end of March when lockdown started, here the words are more present tense and stronger eg struggling, alone. However irrespective of the peaks all words have reduced since the end of March as our users have got used to ‘Lockdown life’, although their usage has stayed consistently higher than before lockdown. |
| **The Mighty**  Insights based on a weekly survey to Mighty members. We have collected around 45K responses over 5 weeks. About 20-25% of those identify as having a mental health condition.   - Impact to healthcare is rising - 79% of those with a mental health condition say their access to healthcare has been impacted (up from 57% four weeks ago) - 72% have missed regularly scheduled appointments, and 24% are having difficulty accessing medications or treatments - 29% say their condition has worsened (up from 15% four weeks ago) - 89% say their daily life has been at least somewhat impacted by increased anxiety; 43% say it has been extremely impacted - 45% say their relationships have become more strained overall; 26% say they have become stronger. The oop reasons for relationships becoming stronger are spending more time with loved ones while top reasons for strain is inability to leave home or see loved ones - The community reports long wait times or lack of available of telehealth appointments as a major struggle in getting the care they need - The community feels their mental health conditions are deprioritized by the healthcare community due to a focus on COVID-19 |
| **TalkLife & TalkCampus**   - In the UK, there has been a 20% increase in monthly active users since COVID19 began. - In terms of engagement on the platform during this period, we have seen a 25% increase in content across comments, posts, and messages. |
| **Wisdo**   - 283% increase in the number of people daily replying to other people's messages in groups (i.e, increases in the engagement around people answering other people in groups.) - +100% increase in the number of people daily joining more group communities - +75% increase in the number of searches daily - +53% increase in the number of "Instant Chat" requests, meaning people looking to instantly connect to someone 1:1 who's "been there", for support - +20% increase in the number of people watching user generated videos, to get them through the day - +115% increase in the number of people weekly willing to step up, become "Wisdo Helpers" and be there for others. - Average daily sessions per user have risen from a high of 3 a day on av. pre Corona, to 7 a day on av. now. - Graphs and week-by-week trends can be found here (page 5 onwards): <https://docs.google.com/document/d/1yiWmlZORI0uMlrYwUMoc7ic64PGEb1mUW6GXsIUuf64/edit> |
| **MeeTwo**  ● There were 27 suicidal posts between 8.30am and 8pm on 22^nd^ March 2020 (48 hours after schools were closed), as compared to 406 suicidal posts in all of 2019.  ● There was a 95% increase in level 4 (severe risk) between 20th March and 4th April 2020, as compared to 20th December 2019 and 4th January 2020.  ● 116% increase in level 3 (high risk) posts between 20th March and 4th April 2020, as compared to 20th December 2019 and 4th January 2020. |
| **MIELI Mental Health Finland**  **Mental-chat**   - Data gathered by the staff / volunteers who work on Mental-chat - After February, we have trained 267 new professionals and 88 volunteers to work on Mental-chat - Comparison between January - March 2019 and January - March 2020 shows 13% increase on dropped chat requests (they don´t get through due to massive queue), and 25% increase on chat conversations. - Based on the 2 first weeks of April we are going to make a new record of chat conversations in a month (in 2 weeks already 1870 conversations) - COVID-19 has been discussed in 7% of the conversations. Young people have been mostly concerned about school, their mental health during lockdown at homes, concerned about loved ones (grandparents f.ex.). Also many of them feel concerned about their mental health while offices are closed, therapy sessions cancelled or moved to online which does not suit everyone. Other significant topics (1st Jan - 19th April 2020): feeling bad (includes anxiety, feeling depressed, eating problems), 26%; relationship issues, 13%; problems with everyday life, 11%; suicidal thoughts, plans, attempts, 10%   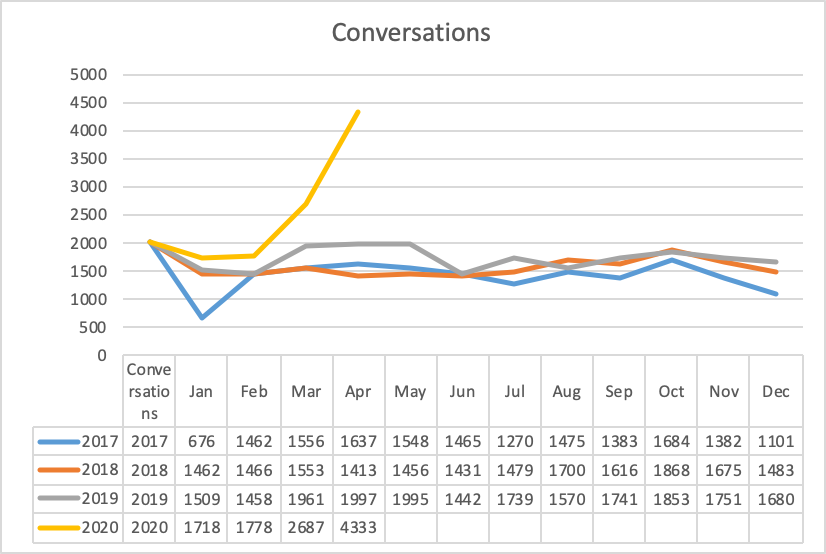  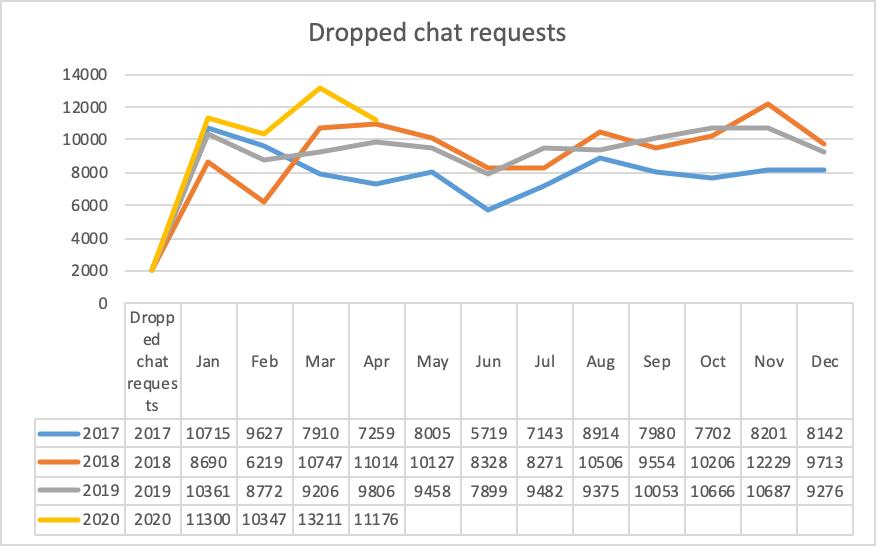    **Mental Gaming**   - We opened #korona channel to our Discord server and will keep it open as long as necessary (first week 1478 messages, after that 45-144 per week) - #mentalhealth channel has been hugely popular throughout Covid-19 crises - Increase of members +821 within 16th March - 29th March 2020. After that, almost 500 new members per week. - All together there are 13,064 members on our server (in April) (the biggest Discord server in Finland)   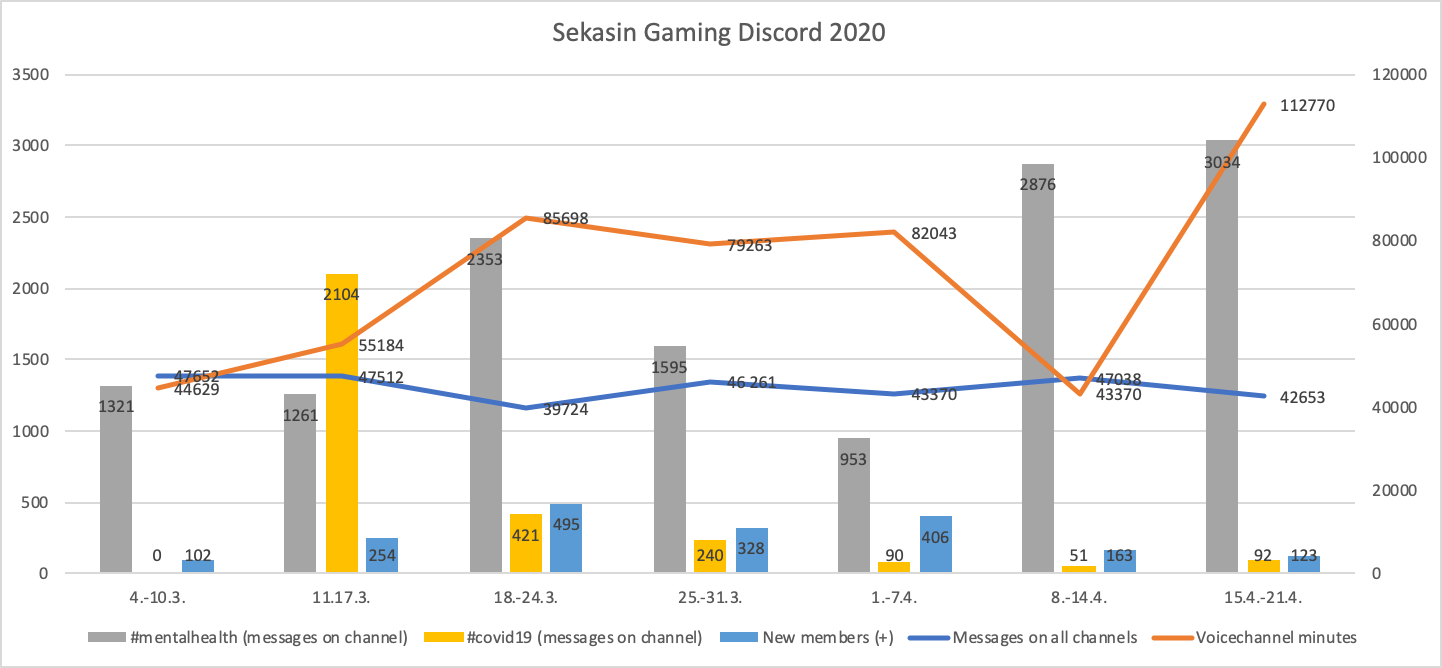 |
| **Teen Line**  Insights based on hotline data (text and emails only with remote hotline) analyzed from 13th March 2020 - 2nd May 2020 and compared to the same time period in 2019:  ● Increase of reports of child abuse by 166%, stress and anxiety by 20%, and loneliness by 13%.  Insights based on the number of active users of Teen Line message boards, an online community of over 38,000 youth worldwide. Data reflect the time period of March 13-April 20, 2020:   - As compared to the same time period in 2019, there was a 29% increase in the number of youth visiting Teen Line’s message boards.   Insights based on comparison of Feb 2020 to March 2020 for Teen Talk, peer support app, with over 28,000 users:   - 55% increase in monthly active users - 50% increase in new users |
| **Papa**  When our members (elderly) initially enroll into our program, we screen them using scales shown below. After 6 months of usage we reassess our members using the same measure:  The UCLA's Loneliness Scale: These results are scored and categorized into three levels of loneliness: "not lonely", “lonely”, and “severely lonely”. In our most recent analysis, we found:   - Loneliness impact: On average, 53% of those using our service felt less lonely (in other words, scored lower on the UCLA loneliness scale   We use the CDC's "Healthy Days Measure" to assess physical and mental unhealthy days. This measure is a 4-item set asking participants to quantify the amount of days they felt physically unhealthy and mentally unhealthy in the past 30 days:   - When we analyzed our impact on mentally/physically unhealthy days, on average, our members' unhealthy mental days lessened by 14% and unhealthy physical days lessened by 16%.   *We perform monthly updates/analysis of our members. This analysis was performed last month for Early March and beyond.* |
| **Digital Peer Support**   - there has been a 50% increase in the amount services being provided digitally for digital peer support - From 10 March 2020, 750 Peer support specialists have been trained in digital peer support (digitalpeersupport/certification) and they are able to leave their virtual doors open. The certification is associated with increased confidence and capacity to use digital peer support (see qualitative section for more details). |
| **National Alliance on Mental Illness (NAMI)**  **NAMI Helpline:**   - Since COVID-19, NAMI has experienced an increase in demand for online/virtual mental health support and resources. Looking at year-over-year NAMI HelpLine inquiries for the period covering March 1 – April 23, during this time in 2019 there were 5,103 calls, call backs and emails compared to the same time in 2020 which had 7,191 calls, call backs and emails. This represents a 41% increase in demand for HelpLine resources and information.   **NAMI Basics OnDemand (launched in October 2019):**   - We provide parents with access to the resources and in January 2020, we had 512 registrations, whereas in just the first week of April 2020 (4/1-4/8) we had 3,481 registrations. Average NAMI Basics OnDemand registrations per day: 17 in January: 435 in April. |
| **Mental Health America**   1. **General Anxiety Disorder-7 (GAD-7):** The number of GAD7 anxiety screens taken in March 2020 was 22% higher than February 2020, and about a 25% increase over the average number of anxiety screens over November 2019-February 2020. The pace of anxiety screenings in April has increased even more. The average per day number of anxiety screens increased 67% since January 2020, reaching 513 screens per day in the first half of April (4/1-4/15). 45% (N=4,994) of people who took an anxiety screen in March (N=11,033) scored for **severe anxiety**. In the first 15 days of April 2020, 44% (N=3,366) scored for severe anxiety. 2. **Pediatric Symptom Checklist-35 (PSC-35):** There was a 20% increase from Q4 2019 to Q1 2020 (N=7,313 in Q1) in people taking the youth screen (PSC-35). The percentage of youth screening at risk for emotional, attentional or behavioral difficulties on the PSC-35 has increased every month since October 2019. In March, 76% (N=2,172) of youth who took the youth screen scored at risk. This was a 5% increase from February 2020 (N=1,652), and a 10% increase over the average rate of at-risk screens in Q4 2019 (N=4,023). 3. **Patient Health Questionnaire-9 (PHQ-9):** There was an 18% increase in the number of PHQ-9 depression screens between March 2020 (N=16,481) and February 2020 (N=13,954). The percentage of people who scored moderate to severe depression on the PHQ-9 increased 3% in March 2020 (to 83%, N=13,745) compared to February 2020 (80%, N=11,151). (i.e., both the number of screens increased and the rates of severe screens increased, indicating that more people were coming to look for mental health screening and when they screened, more people were screening as severe.) We thought people were taking more screens because of coronavirus when the screens increased in February, but in April we added the coronavirus question and it confirmed what we speculated, which is that isolation and coronavirus are major challenges that are bringing people to screening. 4. In response to the question “What are the main things contributing to your mental health problems right now? Choose up to three,” the main reported concern among people taking an anxiety screen was loneliness or social isolation (58% of respondents, N=701). The second most common concern was coronavirus (48%, N=572). 5. On March 16^th^, Mental Health America launched a covid resource page that is updated daily to provide information, resources and support to the mental health community at <https://www.mhanational.org/covid19>. 106,985 users viewed the content since it launched with an average of about 20k users a week. |
| **Mental Health Foundation**  Polling of over 2000 UK adults aged 18+ (weighted to be representative of UK adult population) once before UK restrictions (t1: 17-18 March) and again just after (t2: 2-3 April) *All figures, unless otherwise stated, are from YouGov Plc:*   - People reporting they felt lonely up from 10%(t1) to 24%(t2) - Most pronounced for people aged 18-24 16%(t1) to 44%(t2) and 25-34 14%(t1) to 35%(t2) - 26% reported the pandemic was “making my existing mental health problems worse” (but no change between t1 and t2) - 65%(t2) reported being worried about becoming ill with the virus (58% at t1) - 54%(t2) reported feeling panicked or afraid (52% at t1) - 65%(t2) reported feeling anxious or worried (62% at t1) - When asked about coping with stress (only asked at t2) - 20% reported drinking more alcohol (highest in the 35-44 age group 28%, higher in social groups ABC1 (23%) compared to C2DE (16%)) - 7% reported smoking more (highest in age 25-34 (10%) higher in social groups C2DE (9%) compared to ABC1 (5%) - 30% reported eating too much (women 35%, men 24%, people aged 18-24 42%) |
| **Mentally Aware Nigeria Initiative (MANI)**   - 40% increase in numbers of people who reached out to MANI for psychosocial support and counselling in the month of February alone (based on the total number of cases handled in March). - From March to April, there has been a 55% increase in the number of cases handled (based on the total number of cases from the month of March). - Recorded the highest number of emergency calls in the month of April. 26% of these calls were cases of panic attack, 8% bordered around incessant low moods and the rest (66%) were suicidal. - 40% of people who have reached out to us in the past month (April) via our social media channels were seeking help on behalf of a spouse, friend or relative. - 200+ trained psychosocial support team of specialists (counsellors) on mental health - 3 emergency contact service (numbers) available for support |
| **Money and Mental Health Policy Institute**  Respondents reported a range of concerns about how changes as a result of coronavirus might affect their finances:   - 62% - worried about having to access the benefits system - 57% - worried about losing their job - 56% - worried about creditors chasing them for money |
| **Qntfy**  Qntfy has a collection of users who work on the front lines of the COVID-19 pandemic as healthcare professionals (n=25,046), and a sample of the general population (n=10,000) -- all of whom speak English and have posted at least 100 messages on social media since March 1, 2020. For all these users, we have public social media data going back at least into 2019. Qntfy's models for anxiety, depression, and suicide risk are used here to score the language of each message from the users in the cohort -- these were created via similar methodology as (ref 11), but have significantly more users and improved machine learning techniques applied). A composite well being score is derived from these individual message-level via proprietary methods, but can be generally understood as follows:   1. Each message was scored by each model, producing a single score per message. 2. Message scores were aggregated across models and over short windows of time (order days) to produce estimates of wellbeing per day per person.   This plot shows the average well being score per day for each of these cohorts. Both cohorts have decreased since the onset of COVID-19 and the associated lockdowns (indicated here by a vertical gray line). While the wellbeing of the general population has decreased, the wellbeing of the healthcare providers has decreased more. Omitted for brevity are similar phenomena for the individual results from Qntfy's depression, anxiety, and suicide risk models independently.  **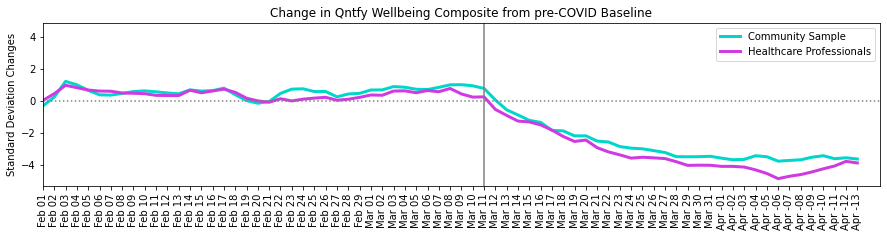** |
| **Practicing NHS clinician in North West London and Dorset specialising in general medicine: Insights provided by Jasvinder Kandola.**   - In a patient population of 14,000 patients there has been a 64% increase in the number of consultations relating to stress, anxiety and low mood since social isolation / distancing was introduced by the UK government. - A retrospective analysis of anonymised patient records revealed 97% of all patients cited Covid-19 and social isolation as being the main contributing factors to their decline in mental health. - There has been a 69% increase in consultations related to mental health where patients have never consulted about mental health issues before (stress, anxiety, depression) - In the period 1st March - 18th April 2020 there has been an 83% increase in the prescription of first line anti-depressant medications as per NICE guidelines across all age groups. - In the over 80s age group there has been a 91% increase in prescription of anti-depressant medication - 0.6% of the over 70s admitted making use of the UK government isolation helpline |
| **Google trends (Hannah Stewart):** <https://trends.google.com/trends/explore?hl=en-US&tz=-330&geo=US&q=coronavirus+anxiety&sni=6>   - ‘Interest’ for the term “anxiety symptoms” doubled globally between the weeks beginning 8 March and 22 March 2020. - The week beginning 22 March 2020, the search term reached a new five-year peak. In Spanish speaking countries, that peak came a week sooner. Conversely, interest for “depression symptoms” fell during the same time period. - ‘Interest’ in “panic attack symptoms” has nearly doubled from 16 February to 22 March 2020. Like “anxiety symptoms”, “panic attack symptoms” reached a global five-year high during the week beginning 22 March 2020. - ‘Interest’ for the term ‘meditation’ has grown in popularity and ‘online CBT’ has increased in recent weeks whereas ‘suicidal thoughts’ and ‘anorexia’ *appear* to have decreased. - "OCD symptoms" hit a global peak in "interest" the week beginning 19 April 2020 (this past week) and has been on the rise since 22 March 2020. - "OCD" has been on the rise since the week of March 15 and is at a current "interest" of 90 (essentially measured as 90% as popular as it's highest search interest ever). "Obsessive-compulsive disorder" is at its global peak and has been trending up since March 15. - "Managing OCD" has also jumped up over the month of April, but Google says the data is incomplete. "Managing anxiety" trended up starting the week of 2/16 and hit its peak the week of 3/15. It's been trending downward since then. |
| **Turn2us**  Turn2us information on mental health of service users based on 6,198 survey responses from users of benefit calculator and 250 responses from grant applicants, since 16 March 2020  **ONS4 Wellbeing questions:**   - 47% reported 'high' anxiety levels - an increase from 38% pre-Covid - 36% reported low 'life satisfaction' - an increase from 34% pre-Covid - 28% reported low levels of feeling life was worthwhile (4 or below on an 11-point scale) - no change since pre-Covid - 41% reported low levels of happiness (4 or below on an 11-point scale) - an increase from 36% pre-Covid   **Ability to pay for rent and mortgage:**   - Currently, large proportions are unable to afford rent and mortgages, as a result of coronavirus and its impact on employment. - On benefits calculator, 58% of users who have had employment affected are unable to afford rent or mortgage payments, compared to 34% of users who have not seen work affected by covid - On our grants programme, 70% of users who have had employment affected are unable to afford rent or mortgages, compared to 30% of users who have not seen work affected - Ability to pay for housing, and the fear of homelessness (seen a lot in the qualitative responses) is probably a factor in the stress and anxiety that people on low incomes are experiencing in the UK - There are no differences seen in users' ability to afford food or heating, due to coronavirus affecting employment, although the % is still relatively high. 30% of all users are currently unable to afford food and 33% are unable to afford heating or electricity |
| **Anonymous Financial Services Provider:**  One large lender/financial services organisation reported:   - Details to be inserted once payroll analysis completed end of April |
| **Tully and OpenWrks Group**  This sample data is formed of people (N=1822) who have selected to come to Tully for Covid-19 financial and well-being support:     - 50% of people’s income has reduced due to COVID-19. A reduced income includes furloughed, reduced hours and reduced pay. - 19% have lost their income due to COVID-19. Lost income includes redundancy and unpaid leave. - From a subset of 650 self-employed customers:   - 80.7% have declared that they don’t have any work coming in due to COVID-19.   - 12.15% have a reduced workload due to COVID-19. |
| **The Mind and Soul Foundation**  The Mind and Soul Foundation created a web report from their website showing the number of page requests between November 2019 to today (see Figure and Table below). Articles that reference Covid and faith have been particularly read and on average receive a x4 hit rate to our usual readership.  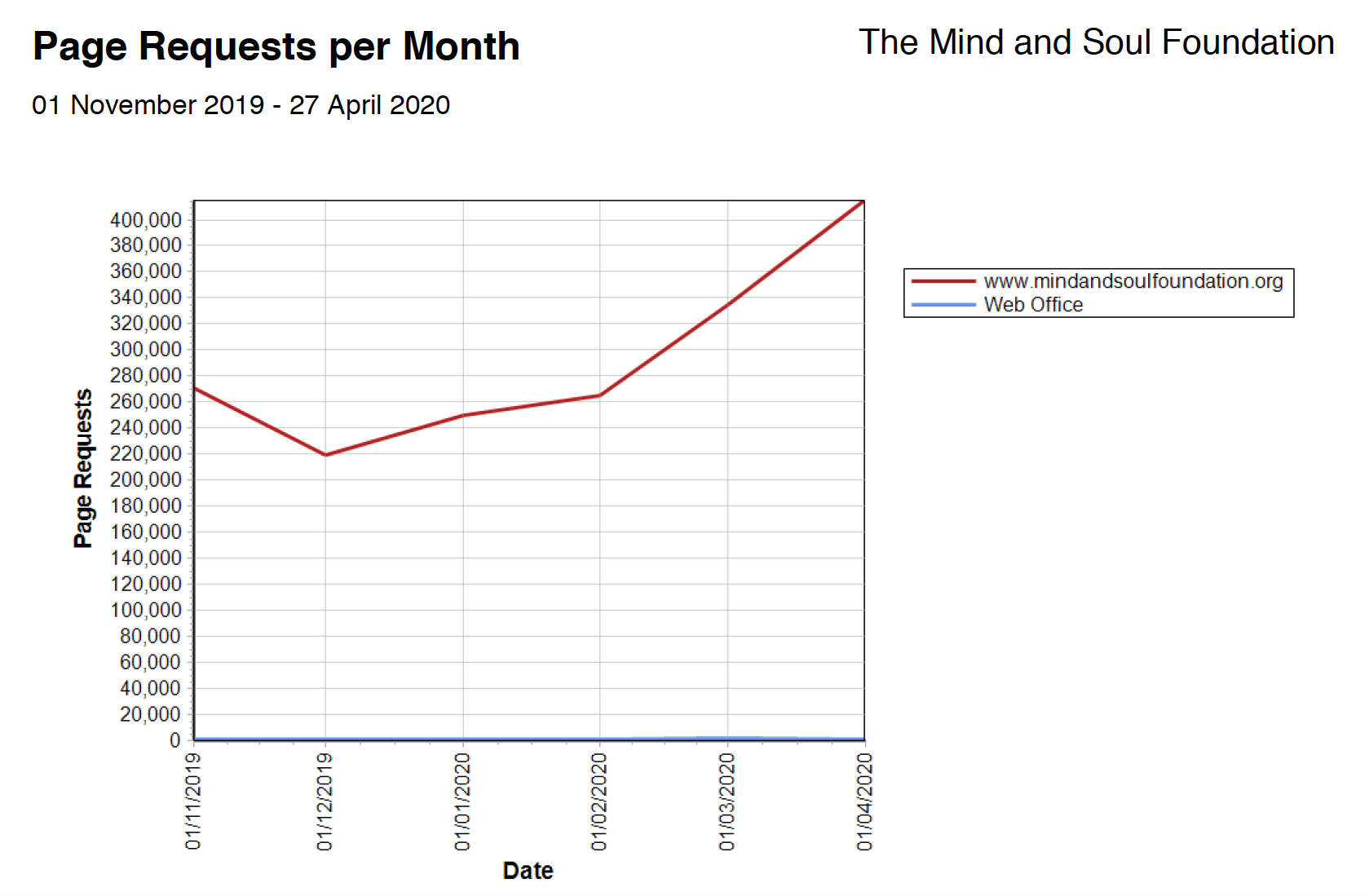 |
| **Darknet Markets Analysis, with some caveats and anecdotes (The TellFinder Alliance)**  We examined two questions (asked by BI):   1. “I suspect… that we might start to [see] increases in opioid drug sales and other mental health-related drug categories like anxiolytics.” 2. “Are people selling covid solutions on the dark net markets?”   **TellFinder dataset details:**  The dataset used to identify high-level insights for the above questions include advertisements for goods and services posted on darknet websites. Specific insights related to effects of the pandemic on the market range from mid-January of 2020 to mid-April. Please note that complications with data retrieval/cleaning may be seen through “spikes” and are acknowledged within the analysis. Because these spikes are addressed within the analysis, overall trends should be treated as an accurate reflection of the dataset.  Within the TellFinder DNM collection, we’ve seen an overall increase in advertisement postings in the last 2 months (Feb.15.2020 - mid April).  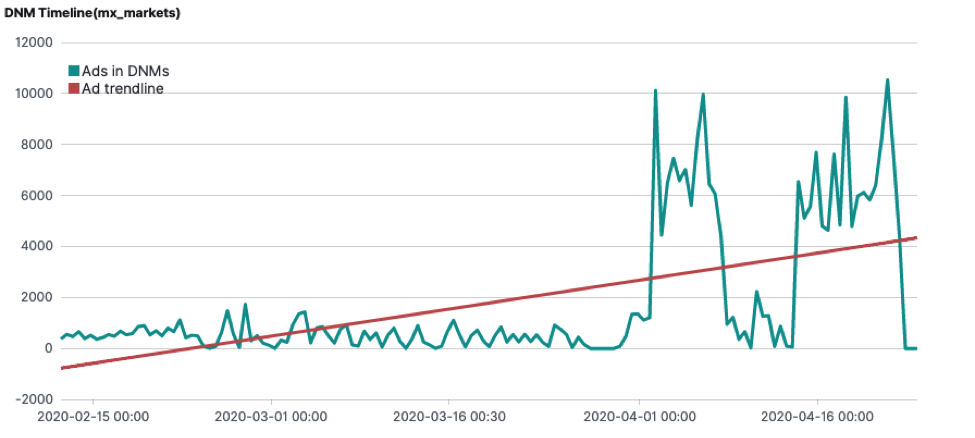  The same collection has also seen an increase in advertisements that mention anxiolytic” since the beginning of April.  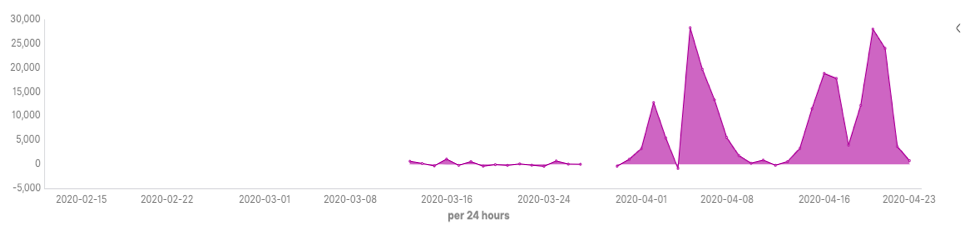  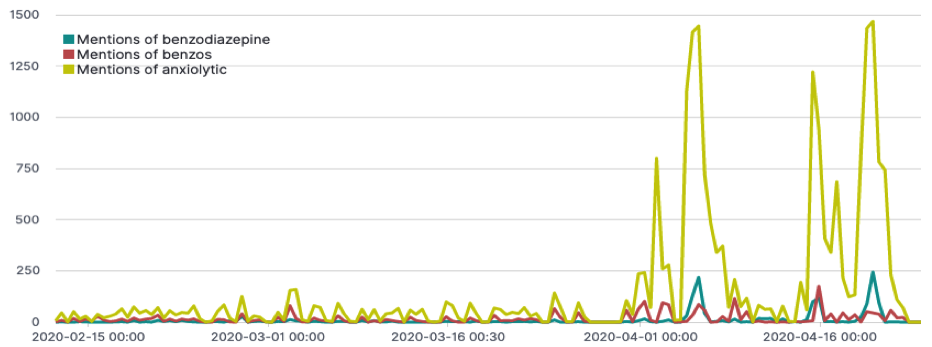  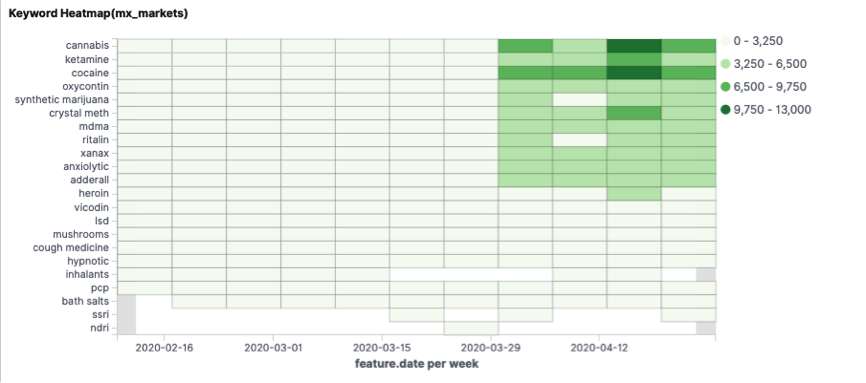  We’ve also observed an increase in new Tor websites (aka “.onion” urls) with advertisements for ”anxiolytic” mentions since the beginning of April.  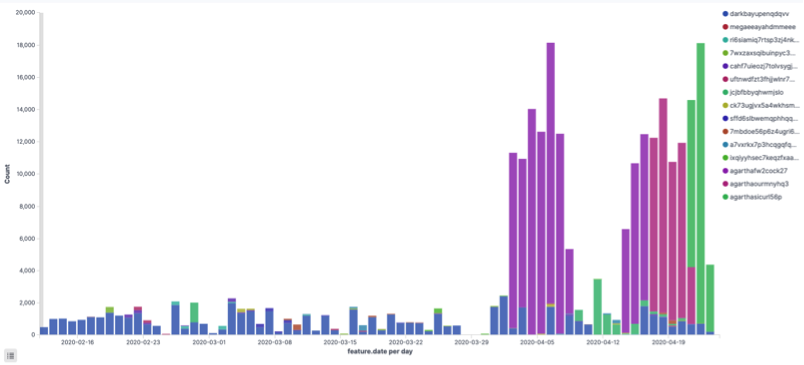  **Summary of Findings:** We have observed a marked increase in anxiolytics roughly associated with the COVID-19 pandemic timeline. We also have an increase in ad *collection* that corresponds with the same period. We have to be careful not to conflate this finding with an uptick in ads more generally, but even holding out the new collectors we still see an upward trend in anxiolytics.  **Anecdotes:** There are new advertisers within the space mentioning drugs, including “tested and trusted” vaccines for the virus, and advertisements for suicide assistance drugs, which do not adhere to the Samaritan's media guidelines about not describing or naming methods of suicide <https://www.samaritans.org/about-samaritans/media-guidelines/> |
| **Healthy Virtuoso**   - the average steps per user has drastically dropped to an average of 2000 steps per day per person. Can we compare this to before COVID19 somehow either compared to this time last year? - The Average number of sports that have been practiced has drastically dropped due to the impossibility of going out - Is there a number or percentage we can add, compared to this time last year? |
